# Biological Determinants of Chemo-Radiotherapy Response in HPV-Negative Head and Neck Cancer: A Multicentric External Validation

**DOI:** 10.3389/fonc.2019.01470

**Published:** 2020-01-10

**Authors:** Martijn van der Heijden, Paul B. M. Essers, Monique C. de Jong, Reinout H. de Roest, Sebastian Sanduleanu, Caroline V. M. Verhagen, Olga Hamming-Vrieze, Frank Hoebers, Philippe Lambin, Harry Bartelink, C. René Leemans, Marcel Verheij, Ruud H. Brakenhoff, Michiel W. M. van den Brekel, Conchita Vens

**Affiliations:** ^1^Division of Cell Biology, The Netherlands Cancer Institute, Amsterdam, Netherlands; ^2^Department of Head and Neck Oncology and Surgery, The Netherlands Cancer Institute, Amsterdam, Netherlands; ^3^Department of Radiation Oncology, The Netherlands Cancer Institute, Amsterdam, Netherlands; ^4^Amsterdam UMC, Vrije Universiteit Amsterdam, Otolaryngology/Head and Neck Surgery, Cancer Center Amsterdam, Amsterdam, Netherlands; ^5^Department of Radiation Oncology (MAASTRO), GROW—School for Oncology and Developmental Biology, Maastricht University Medical Centre, Maastricht, Netherlands; ^6^The D-Lab and The M-Lab, Department of Precision Medicine, GROW—School for Oncology and Developmental Biology, Maastricht University, Maastricht, Netherlands; ^7^Department of Radiation Oncology, Radboud University Medical Center, Nijmegen, Netherlands; ^8^Department of Oral and Maxillofacial Surgery, Amsterdam UMC, Academic Medical Center, Amsterdam, Netherlands

**Keywords:** HNSCC, chemoradiotherapy, radiation resistance, hypoxia, immune cell infiltration, expression profile analysis, head and neck cancer, radiation oncology

## Abstract

**Purpose:** Tumor markers that are related to hypoxia, proliferation, DNA damage repair and stem cell-ness, have a prognostic value in advanced stage HNSCC patients when assessed individually. Here we aimed to evaluate and validate this in a multifactorial context and assess interrelation and the combined role of these biological factors in determining chemo-radiotherapy response in HPV-negative advanced HNSCC.

**Methods:** RNA sequencing data of pre-treatment biopsy material from 197 HPV-negative advanced stage HNSCC patients treated with definitive chemoradiotherapy was analyzed. Biological parameter scores were assigned to patient samples using previously generated and described gene expression signatures. Locoregional control rates were used to assess the role of these biological parameters in radiation response and compared to distant metastasis data. Biological factors were ranked according to their clinical impact using bootstrapping methods and multivariate Cox regression analyses that included clinical variables. Multivariate Cox regression analyses comprising all biological variables were used to define their relative role among all factors when combined.

**Results:** Only few biomarker scores correlate with each other, underscoring their independence. The different biological factors do not correlate or cluster, except for the two stem cell markers CD44 and SLC3A2 (*r* = 0.4, *p* < 0.001) and acute hypoxia prediction scores which correlated with T-cell infiltration score, CD8^+^ T cell abundance and proliferation scores (*r* = 0.52, 0.56, and 0.6, respectively with *p* < 0.001). Locoregional control association analyses revealed that chronic (Hazard Ratio (HR) = 3.9) and acute hypoxia (HR = 1.9), followed by stem cell-ness (CD44/SLC3A2; HR = 2.2/2.3), were the strongest and most robust determinants of radiation response. Furthermore, multivariable analysis, considering other biological and clinical factors, reveal a significant role for EGFR expression (HR = 2.9, *p* < 0.05) and T-cell infiltration (CD8^+^T-cells: HR = 2.2, *p* < 0.05; CD8^+^T-cells/Treg: HR = 2.6, *p* < 0.01) signatures in locoregional control of chemoradiotherapy-treated HNSCC.

**Conclusion:** Tumor acute and chronic hypoxia, stem cell-ness, and CD8^+^ T-cell parameters are relevant and largely independent biological factors that together contribute to locoregional control. The combined analyses illustrate the additive value of multifactorial analyses and support a role for EGFR expression analysis and immune cell markers in addition to previously validated biomarkers. This external validation underscores the relevance of biological factors in determining chemoradiotherapy outcome in HNSCC.

## Introduction

In this study we set out to perform multifactorial analyses to gain understanding of the role and dependence of biological factors that have shown to influence tumor radiation response in preclinical studies and to be associated with radiotherapy response in clinical studies (1, 2). Chemo-radiotherapy is the primary treatment option for advanced head and neck squamous cell carcinoma (HNSCC). Cure and locoregional failure rates of around 50 and 25%, respectively, facilitate the evaluation of biological determinants of radiation response. Using biological characteristics of the tumors, outcome association studies revealed many potential determinants of prognosis and treatment response in HNSCC (3–7). This study evaluates complementarity and hierarchy of radiation response determining “HNSCC biology.”

Head and neck squamous cell carcinoma (HNSCC) is the 6th most common cancer in the world, with smoking, alcohol and HPV infection as the main risk factors. Around two thirds of the patients present with advanced stage disease and have a poor prognosis with 5 year overall survival rates around 50% (8, 9). Allowing for organ preservation, curative treatment for advanced stage hypopharyngeal, laryngeal and HPV-negative oropharyngeal carcinomas shifted from extensive surgery to concomitant cisplatin-based chemo-radiotherapy in the last decades (10). Around two third of all HNSCC patients receive radiotherapy as part of their treatment. Among these, those with HPV-positive oropharyngeal tumors have a particularly good prognosis, reason to consider them as a new entity in the new TNM staging (11). As revealed by gene expression and mutational analyses, these tumors are also biologically very different (12, 13). HPV-negative HNSCC, in contrast, are characterized by poor prognosis. They exhibit frequent amplifications and mutations in proto-oncogenes (EGFR, MYC, HRAS) and in cell cycle genes that drive and support tumor proliferation (14–16). p53 is affected in almost all HPV-negative HNSCC.

Early radiobiology studies revealed determinants of tumor radiation response. Hypoxia, repopulation, driven by tumor cell proliferation, tumor stem cell density (i.e., clonogenic cell density) and cellular radiosensitivity (as for example determined by cellular DNA damage repair capacity) were shown to be among the most relevant biological factors that affect radiation or fractionated radiotherapy response in preclinical models of different cancers (1, 2). In recent years, increased interest emerged in immune response related markers and immune cells due to novel immunotherapeutic options (17–21). A series of preclinical and clinical studies highlight the potential relevance of immune-related markers in HNSCC [reviewed in (5, 6, 19, 22–24)].

HNSCC outcome association studies using many different biomarkers, demonstrated the clinical importance of some of these pre-clinically assessed tumor biology parameters (1, 5–7). HPV and hypoxia are indeed the best studied biology related prognostic markers in HNSCC. Within the HPV-negative patients, tumor hypoxia marks patients with a poor prognosis (25–29). Confirming its role above marking poor prognosis patients, hypoxia biomarkers also predict response to hypoxia modification therapy (25, 30–33). Elaborating on a gene expression profile that captures the cellular changes caused by acute hypoxia, we recently showed the relevance of acute hypoxia in addition to chronic hypoxia (29). As predicted by the process they capture, these two classifications did not necessarily overlap in the samples and also reveal different outcome associations in HNSCC that result from a prominent role of acute hypoxia.

While the success of accelerated radiotherapy schedules (34) highlight the important role of tumor repopulation in HNSCC, there is a lack of biomarker data showing a link to cellular proliferation (35). Based on genetic mutation data, we find a small role for co-occurring CCND1 and CDKN2A mutations in HPV-negative chemo-radiotherapy treated HNSCC that was however not visible in the locoregional control endpoints (36). Yet, the combination of radiotherapy with the epidermal growth factor receptor (EGFR) binding antibody cetuximab has shown efficacy and EGFR expression has been associated with poor survival, preferentially in non-accelerated schedules arguing for a role in tumor repopulation (37–40). The role of EGFR and cellular proliferation in radiotherapy response needs to be further elaborated (41–43). However proliferation, as determined by the proliferation marker by Starmans et al. has been linked to aggressive disease or disease progression in multiple cancer types; unfortunately this was not assessed in HNSCC (44).

Originated from CD44 expression data from de Jong et al. (45) in laryngeal cancer and confirmed in resected and chemo-radiotherapy treated HNSCC for CD44 and SLC3A2 (27, 46) in subsequent studies, it also became clear that tumor “stem cell-ness” is important in radiotherapy outcomes since these stem cell related biomarkers were associated with poor prognosis (35, 47–49).

The consistent effect of CD8^+^ T cell depletion on radiation induced tumor growth delays in preclinical studies expose the relevance of certain immune cell populations in radiation response and resistance (50, 51). Evidence in clinic of a possible interaction is less strong and current studies focus on strategies to optimize combinations with immune response modulators to improve radiotherapy outcomes (6, 18, 20, 21, 23, 27, 52–55). Interestingly, Mandal et al. recently showed that markers for regulatory Tcells (Treg), NK cells and CD8^+^ T cells are prognostic in head and neck cancer (56) in the TCGA dataset. Despite these interesting initial reports, the prognostic value of these gene expression based immune markers is still unknown for chemo-radiotherapy treated patients since all patients in the TCGA dataset have been treated with primary surgery. Immunohistochemically (IHC) determined high CD8^+^ T-cell counts are associated with good prognosis in postoperative chemo-radiotherapy treated patients, further indicating its relevance for HNSCC (57). A good prognosis association with IHC CD8^+^ TIL density was found in patients with oropharyngeal squamous cell carcinoma treated with surgery or (chemo) radiotherapy and in a similarly mixed treatment cohort of hypopharyngeal SCC patients (58–60).

Our previous studies emphasized the important role of functional and genetic DNA crosslink repair defects in HNSCC (61, 62) and provided the basis for machine learning generated models that predicted such DNA repair defects in clinical samples (63). The expression based DNA repair defect prediction models revealed an association with metastasis in HNSCC and linked DNA repair defects to migratory and invasive behavior in HNSCC cell lines (63). Given the relevance of Epithelial to Mesenchymal Transition (EMT) in many cancer types, we also developed a HNSCC-specific EMT model that classifies HNSCC according to epithelial or mesenchymal characteristics (64). The strong prognostic value of this HNSCC-EMT model also suggests an important role in radiation response.

Taken together, the individual roles of some of these biological factors important in radiation response have not been validated and the interrelation of these biological factors has not been investigated in the clinical setting. We therefore studied the role of the aforementioned biological factors in the context of head and neck cancer and chemo-radiotherapy. Previously published gene expression based signatures were used to detect these factors. In a set of nearly 200 patients with advanced stage HPV-negative HNSCC treated with chemo-radiotherapy, we used univariate and multivariate outcome analyses to examine these factors while also considering correlation and dependence to delineate their relative roles.

## Materials and Methods

### Patient Data and Material

This retrospective study included material and data from patients that were diagnosed between 2001 and 2014 and treated with definitive cisplatin-based chemo-radiotherapy within three centers: the Netherlands Cancer Institute (Amsterdam, NL), the Amsterdam University Medical Center (Amsterdam, NL) or the MAASTRO clinic/MUMC+ (Maastricht, NL). Selection criteria for this gene expression study cohort were (i) concomitant radiotherapy and cisplatin treatment of unresected HNSCC, (ii) hypopharyngeal, laryngeal or HPV-negative oropharyngeal (iii) no prior treatment with chemotherapy or radiotherapy in the head and neck area. To minimize the number of variables, AJCC disease staging, summarizing TNM stage, was used to classify HNSCC patients after determining whether this classification also represented N-staging and its known association with survival well (Supplementary Figure 1). Received radiotherapy regimens were 70 Gy over 35 fractions (up to 77 Gy in ARTFORCE patients) in 7 or 6 weeks (DAHANCA scheme). All patients were treated with either of four different cisplatin regimens: daily [25 × 6 mg/m^2^ Body Surface Area (BSA)], weekly (7 × 40 mg/m^2^ BSA) or 3-weekly (3 × 100 mg/m^2^ BSA) intravenous administration or weekly intra-arterial administration [4 × 150 mg/m^2^ BSA, for 8 patients according to the RADPLAT trial protocol (65)]. Not all patients completed the full chemotherapy scheme. Therefore, cumulative cisplatin doses were calculated and patients were classified into < or ≥ or 200 mg/m^2^ BSA cisplatin, according to literature (66, 67). Survival data was calculated from the start of treatment until the first event was detected. The primary outcome measure is loco-regional control (LRC) and implies absence of recurrences in the radiotherapy targeted regions of the head and neck area. Patient characteristics are provided in Supplementary Table 1. Institutional Review Boards at the Netherlands Cancer Institute, the Amsterdam University Medical Center and the MAASTRO clinic/MUMC+ approved biopsies and collection of fresh-frozen HNSCC tumor material and the use of genetic and clinical data from patients at their respective centers after anonymization. All patients granted written informed consent for biopsy, material use and data use. Pre-treatment tumor biopsy material available for the DESIGN study or collected from the NKI ARTFORCE (68) or RADPLAT trial patients were used for RNA preparation and sequencing. HPV-status of all oropharyngeal carcinomas was determined by immunohistochemical assessment of p16 by a dedicated head and neck pathologist (69) followed by a HPV DNA test on the p16-immunopositive cases and/or confirmed using RNA-sequencing data.

### Material Preparation and RNA-Sequencing

Fresh-frozen tumor samples were sectioned, collected for RNA preparation and in part subjected to tumor percentage evaluation by revision of HE stained coupes by senior head and neck pathologist Dr. S.M. Willems. Only samples with a tumor percentage of >40% proceeded to RNA-sequencing. RNA was isolated using the AllPrep DNA/RNA mini kit (Qiagen). Quality and quantity of total RNA was assessed by the 2100 Bioanalyzer using a Nano chip (Agilent, Santa Clara, CA). Only total RNA samples having RIN>7 were used for library preparation. Strand-specific libraries were generated using the TruSeq Stranded mRNA sample preparation kit (Illumina Inc., San Diego, RS-122-2101/2) according to the manufacturer's instructions (Illumina, Part # 15031047 Rev. E). The libraries were analyzed on a 2100 Bioanalyzer using a 7500 chip (Agilent, Santa Clara, CA), diluted and pooled equimolar into a 10 nM multiplex sequencing pool and sequenced with 65 base single reads on a HiSeq2500 using V4 chemistry (Illumina Inc., San Diego). Reads were mapped against the GRCh38 human genome using TopHat2.1 (70), with options “fr-firststrand,” “transcriptome-index,” and “prefilter multi-hits.” Read counts were determined using HTSeq-count (71) with options “stranded” and mode “union.”

### Expression and Patient Outcome Analyses

All analyses were performed in R 3.4.3 using Rstudio 1.1. Samples were classified and scored for the different analyzed biological characteristics using different gene expression profiles according to the protocols described in the original publication. If not possible due to the lack of original reference data, GSVA, a Bioconductor package for R, was used on raw read counts to calculate gene expression profile scores (72). Transcripts per million (TPM) was used for individual gene expression analyses. Patient outcome analyses were performed using Cox proportional hazard model. Time to event was defined as the time between the first day of treatment and the day the event was detected. Events in the locoregional control data (LRC) were defined by recurrences in the radiotherapy targeted region. Distant metastasis (DM) events were defined by tumors detected outside the head and neck area. A patient's death prior to a possible event led to censoring in the LRC and DM data and no event was recorded. Progression free survival (PFS) was defined as the time from the start of treatment to the day the patient died, had a locoregional recurrence or distant metastasis. Tests were considered significant when *p* < 0.05. A spearman correlation coefficient was computed between continuous variables.

In order to obtain a robust cut-off when transforming a continuous variable into a dichotomous variable we used the bootstrap procedure as described in Linge et al. (28). In brief, 197 sample values were randomly assigned into one bootstrap cohort (from the cohort of 197 patients) while data from the same patient could be chosen multiple times. This procedure was repeated to obtain 10.000 randomized cohorts. At each possible cut-off value of the marker of interest, the individual cohorts were split into a “low” and “high” group and Cox proportional hazards models were fit based on these splits. These models included, next to the newly grouped marker of interest, all clinical variables that were found to be significantly associated with the outcome of interest [Locoregional Control (LRC), Distant Metastasis (DM), Overall Survival (OS) or Progression Free Survival (PFS)]. The fraction of cohorts for which the marker of interest was significantly associated with survival (*p* < 0.05) was recorded for each cutoff. The values of nine adjacent cutoffs were averaged to smoothen the data. The cutoff with the highest fraction of significant associations was chosen for further analysis. Cutoffs that would result in patient subgroups with <10% of the patients were not considered to maintain statistical power. Note that, this analysis was repeated for each endpoint resulting in different cut-offs.

To reduce the number of possible variables included in multivariable analysis we used a backward selection procedure. The most frequent level of each variable was used as the reference level for this analysis. A Cox proportional hazard model was fit containing all biological markers and clinical variables. Then, each individual variable was removed from the model and improvements in model performance by this process were assessed using the Akaike Information Criterion (AIC) from the “stats” package in R. The best model (lowest AIC) was selected for further analysis in the multivariate Cox regression analysis. This process was repeated until removing variables from the model did no longer result in an improved model.

## Results

### Role of Clinical Factors and Patient Characteristics in Chemo-Radiotherapy Outcome

In this retrospective multicenter study, 197 patients met all inclusion criteria and had sufficient tumor material available. All patients were treated with definitive cisplatin-based chemo-radiotherapy for advanced stage HPV-negative oropharyngeal, hypopharyngeal, or laryngeal carcinoma. Patient characteristics are shown in Table 1. The median age in this patient cohort is 62 years and there is a male: female ratio of 3:1. Most patients reported ongoing or a history of alcohol and/or tobacco use. The largest subsite representation is oropharyngeal tumors with 85 patients, then hypopharyngeal with 78 and laryngeal carcinoma with 34 patients. Except for two patients, all patients had stage III/IV classified tumors. As expected, outcomes and survival curves differ according to stage (Supplementary Figure 2). Tumor volume data as determined by delineation on RT planning CT images were available for 166 patients with a median volume of 23.2 cm^3^. Not all patients finished chemotherapy, but 126 patients (63%) received a cumulative dose of and above 200 mg/m^2^ body surface area. Locoregional recurrences occurred in 23.8% (*N* = 49) of cases and distant metastasis in 19.8% (*N* = 39).

**Table 1:** HNSCC patients and tumor characteristics.

**Variable**		**#Patients (total 197)**
Median Age at diagnosis (Range)		61 years (40–80.23)
Sex	Female	55
	Male	142
Alcohol	No	22
	Former alcoholic	22
	Yes	146
	*Missing*	7
Tobacco	Never	5
	Former smoker	30
	Yes	156
	*Missing*	6
Tumor subsite	Larynx	34
	Hypopharynx	78
	Oropharynx	85
Median tumor volume (Range)		23.2 cm^3^ (1.03–752.2)
	*Missing*	31
Stage	IVB	20
	III	40
	II	2
	IVA	135
Cumulative cisplatin dose	<200 mg/cm^2^	67
	≥200 mg/cm^2^	126
	*Missing*	4
Median Follow-up		5.24 years (4.59–5.86)
Locoregional Recurrences		49
Distant Metastasis		39

Clinical factors were tested for their association with locoregional control and other survival outcomes (Supplementary Figure 1, Supplementary Table 1). Consistent with previous reports we find that locoregional control (LRC) is influenced by cumulative cisplatin dose levels (66, 67). The cumulative cisplatin dose of < 200 mg/m^2^ BSA was significantly associated with LRC failure (HR = 2.57, *p* = 0.0012). Female sex shows a trend toward better locoregional control (HR = 0.52, *p* = 0.072). This could however been confounded by the less prominent alcohol consumption characteristics or other differences in lifestyle in this particular patient group. More female patients reported to abstain from alcohol compared to male patients (21.8 vs. 7.4%, *p* = 0.019), which was however not the case for tobacco use (*p* = 0.66). Heavy past or ongoing alcohol consumption was associated with an increased risk for LRC failure (HR = 2.16, *p* = 0.041). Interestingly, age, tobacco, tumor subsite and AJCC stage is not significantly associated with LRC in our patient cohort. The other clinical outcomes (DM, PFS or OS) showed significant associations with sex, tumor volume, stage and cisplatin (Supplementary Figure 1, Supplementary Table 1).

### Tumor Biology Assessment and (in)Dependence in HPV-Negative HNSCC

Preclinical radiobiology studies and clinical biomarker studies exposed many different determinants of radiation response. The number of variables that can be included in statistical analyses are however limited by the cohort size and number of events. Thus, in order to evaluate the relative role of different tumor biology parameters in clinic, we prioritized those with a reported clinical outcome association. The following 12 gene expression signatures were therefore selected to characterize the clinical samples using pretreatment HNSCC transcriptomic data: The Toustrup (1) and Seigneuric (2) expression signatures were used to assess the level of chronic (1) and acute (2) hypoxia, respectively (25, 73). Linked to tumor stem cell richness SLC3A2 (3) and CD44 (4) gene expression values (in TPM) were included since both have been reported to be associated with outcome in chemo-radiotherapy treated patients (74). Economopoulou et al. (5) EGFR expression (in TPM) and (6) the Starmans et al. “proliferation” expression signature (44) were selected as cellular proliferation markers which could influence tumor repopulation between radiotherapy fractions. To cover immune-related factors we further included expression signatures from Senbabaoglu et al. (75) that originated from Bindea et al. (76) and assess (7) ‘T-cell infiltration score' (TIS), (8) CD8^+^ T-cells, (9) CD56^dim^ natural killer (NK) cells abundance while considering the (10) CD8^+^ vs. T regulatory (Treg) cell ratios. This immune status gene expression signature selection is based on the reported outcome association in resected HNSCC (56). Our own studies conducted in HPV-negative advanced HNSCC revealed an important role for EMT and DNA crosslink (CL) repair defects in treatment outcome and these prediction models for mesenchymal characteristics and tumor cell DNA crosslink repair defects (11). “HNSCC-EMT” and (12) “MMConly,” were therefore included in this analysis and are referred to as “EMT” and “DNA CL repair” in this manuscript (63, 64). Most of these biological factors have been tested individually, predominantly in univariable analyses and in different settings in previous studies; however their mutual correlations and possible dependence between them are unknown.

**Figure 1:**
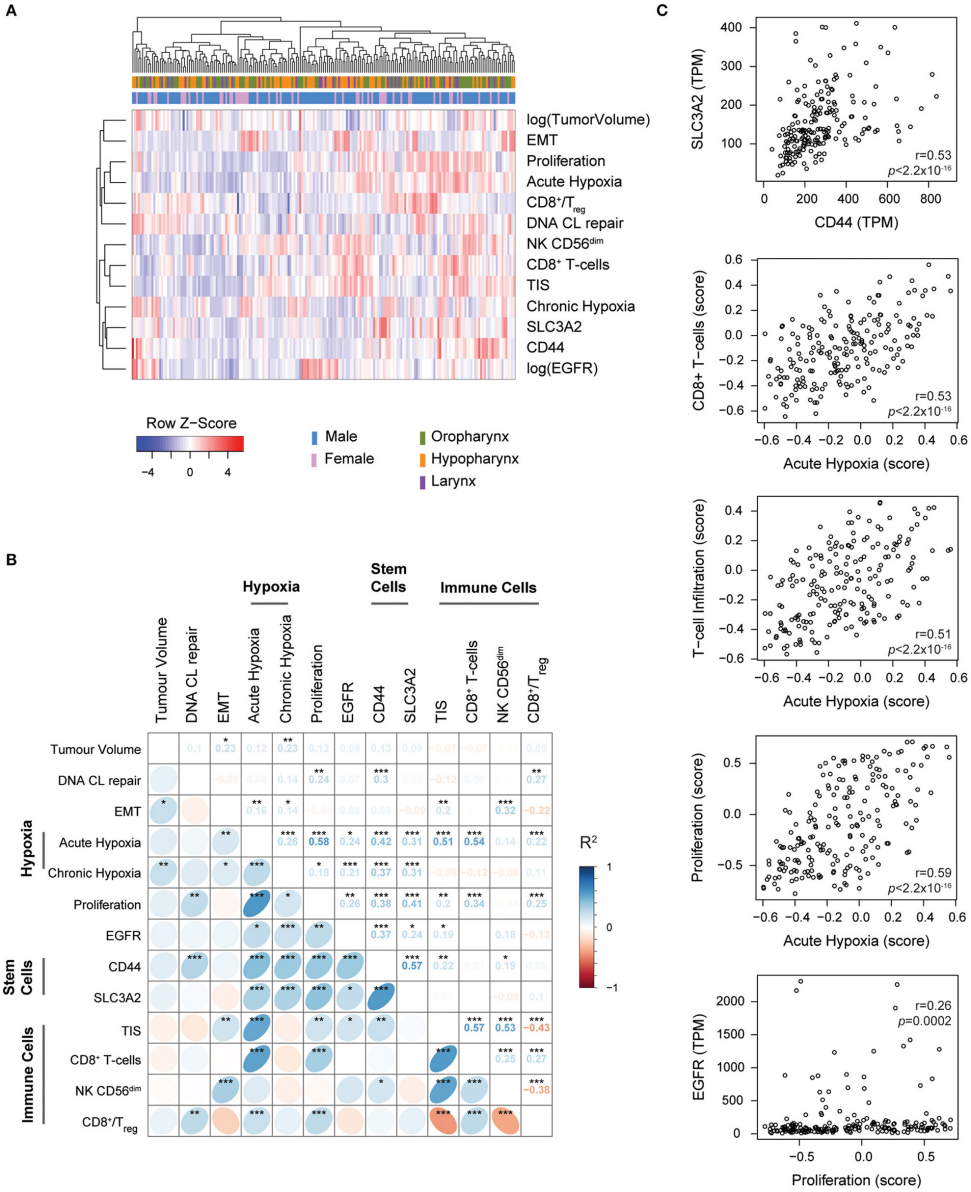
Interrelation and dependence of biological markers. **(A)** Heatmap showing hierarchical clustering of patients based on the selected biological markers. Tumor volume and EGFR expression were log-transformed to prevent clustering to be dominated by few samples with high values. **(B)** Correlation plot with spearman's ranked correlation values between all biological marker scores. Markers were grouped by tumor biology class where appropriate. *p < 0.05, **p < 0.01, *** *p* < 0.001, not corrected for multiple testing. **(C)** Scatterplots with Spearman's coefficients and *p*-values for correlations of interest.

The goal of this study is to pinpoint biological factors that are important for (chemo)-radiotherapy treatment failure and thus might validate their independent role in radioresistance. We therefore calculated scores for all aforementioned markers. Supplementary Figure 3 shows the frequency of the scores and their distribution over the patient cohort. Next, we performed hierarchical clustering to investigate the presence of HNSCC subsets as defined by these characteristics. Tumor volume was included in this analysis as it promotes chronic hypoxia or may be associated with high proliferation scores. Despite the coexistence and correlation of some factors this does however not reveal any prominent clusters (Figures 1A,B). Surprisingly, we find that the acute hypoxia profile score correlates with the Starmans proliferation score (*r* = 0.58, *p* < 0.001) (Figure 1C) but also with the T-cell Infiltration Score (TIS) and the CD8^+^ T cell scores (*r* = 0.51 and *r* = 0.54, *p* < 0.001).

Within their own category, stem cell related markers, CD44 and SLC3A2 (*r* = 0.57, *p* < 0.001), and the immune cell related markers correlate with each other. While the correlation of acute and chronic hypoxia is significant, it is fairly weak (*R* = 0.26, *p* < 0.001) and was in line with previous reports (29). EGFR expression and the proliferation score are correlated to some extent (*r* = 0.26, *p* < 0.01). The CD8^+^ T cell to regulatory T cell ratio (CD8^+^/Treg) as determined by the expression signature scores is negatively associated with the abundance of CD56^dim^ natural killer (NK) cells and the TIS signature. While the link between tumor volume and chronic hypoxia (*R* = 0.23, *p* < 0.01) is expected, tumor volume is also associated with EMT (*R* = 0.23, *p* < 0.05). With a maximum variance inflation factor value of 3.3, correlations were not strong enough to exclude parameters from subsequent analyses. None of these markers show strong associations with any of the clinical factors. Among all, we find that the most independent tumor characteristics are the presence of DNA CL repair defects and tumor EMT status (and tumor volumes).

### Role of Individual Biological Factors in Locoregional Control by Chemo-Radiotherapy in HNSCC

**Figure 2:**
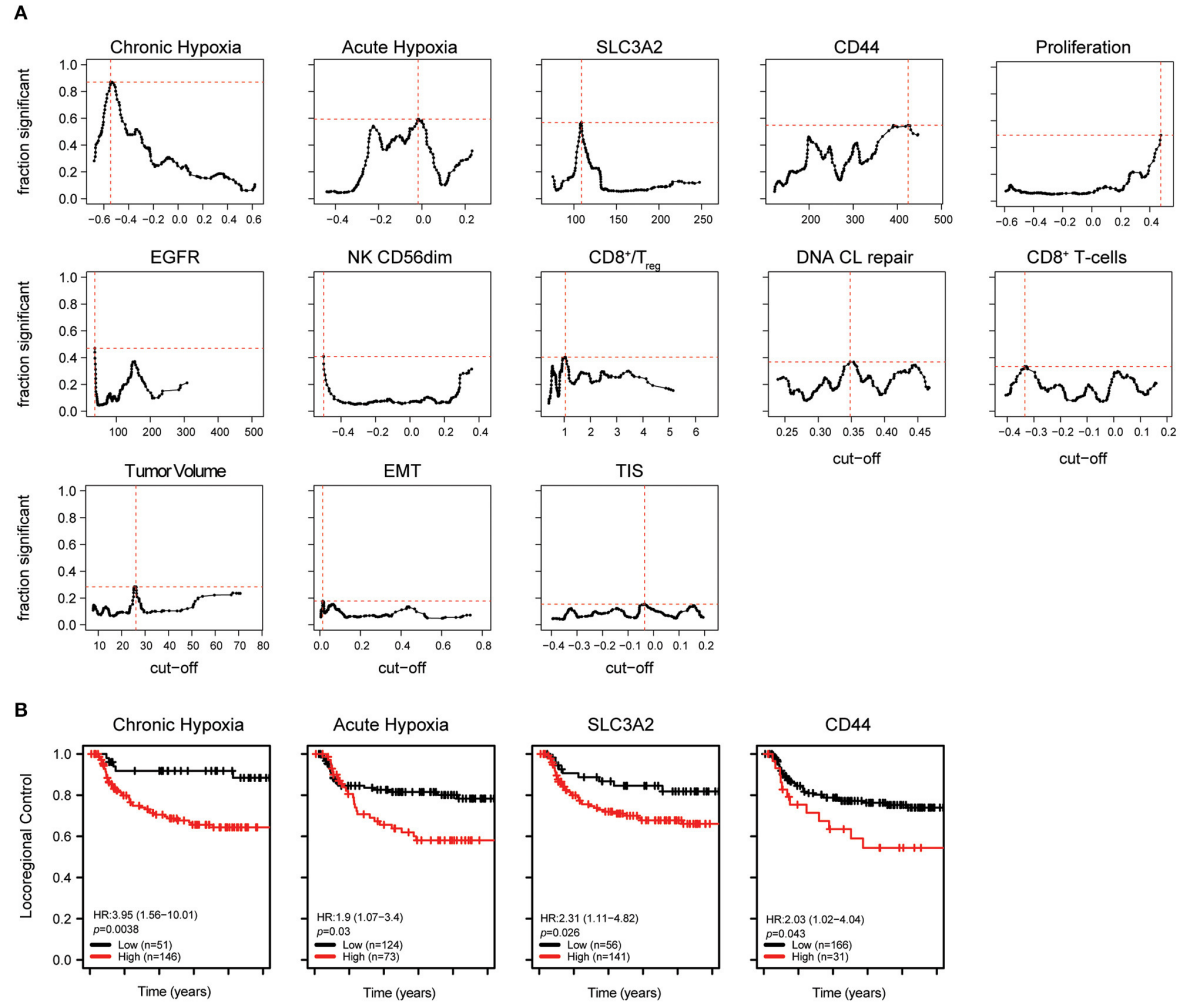
Role of individual biological factors for locoregional control. **(A)** Results of individual bootstrap analysis (see methods) including important clinical factors. The fraction of randomized cohorts with a significant association with locoregional control is shown for each cut-off and biological marker. Markers are ordered by the magnitude of the maximum fraction of significant Cox proportional hazard tests at the best cut-off. The best cutoff is indicated with dotted red lines. **(B)** For each marker, the cohort was split into a high and low group at the best cutoff determined in A. Hazard ratios for recurrences and corresponding *p*-values were obtained with a multivariate Cox proportional hazard analysis using the same variables as those used to determine the cutoff.

Since we aimed to evaluate tumor characteristics with respect to radiation resistance and response, we initially focused on locoregional control outcome values that are mainly determined by the success of the “local” radiotherapy treatment. Given the lack of strong correlations, all markers were individually tested for their association with locoregional failure. A 10.000 times bootstrapping method was employed to (a) determine a potential role for the biomarker across different cutoffs and (b) to identify a clinically robust cut-off for each so to compare the biomarkers among each other. In brief, each marker was tested for their association with the selected survival outcome for all possible cutoffs. This analysis was performed using a multivariable Cox proportional hazard model with all relevant clinical factors included, as determined above. Consequently, clinical variables were included according to outcome type: sex and cumulative cisplatin dose for LRC; sex, subsite and cumulative cisplatin dose for OS; stage, subsite and cisplatin dose for PFS; and sex and alcohol use for DM. Based on the results of these 10.000 bootstrap repeats (Figure 2A), we find that the hypoxia and stem cell related markers are most robustly associated with LRC across different score cut-offs. Proliferation, EGFR and immune cell signatures merely provide significant associations with LRC in a fraction of the randomly created cohorts and tested cut-offs.

**Figure 3:**
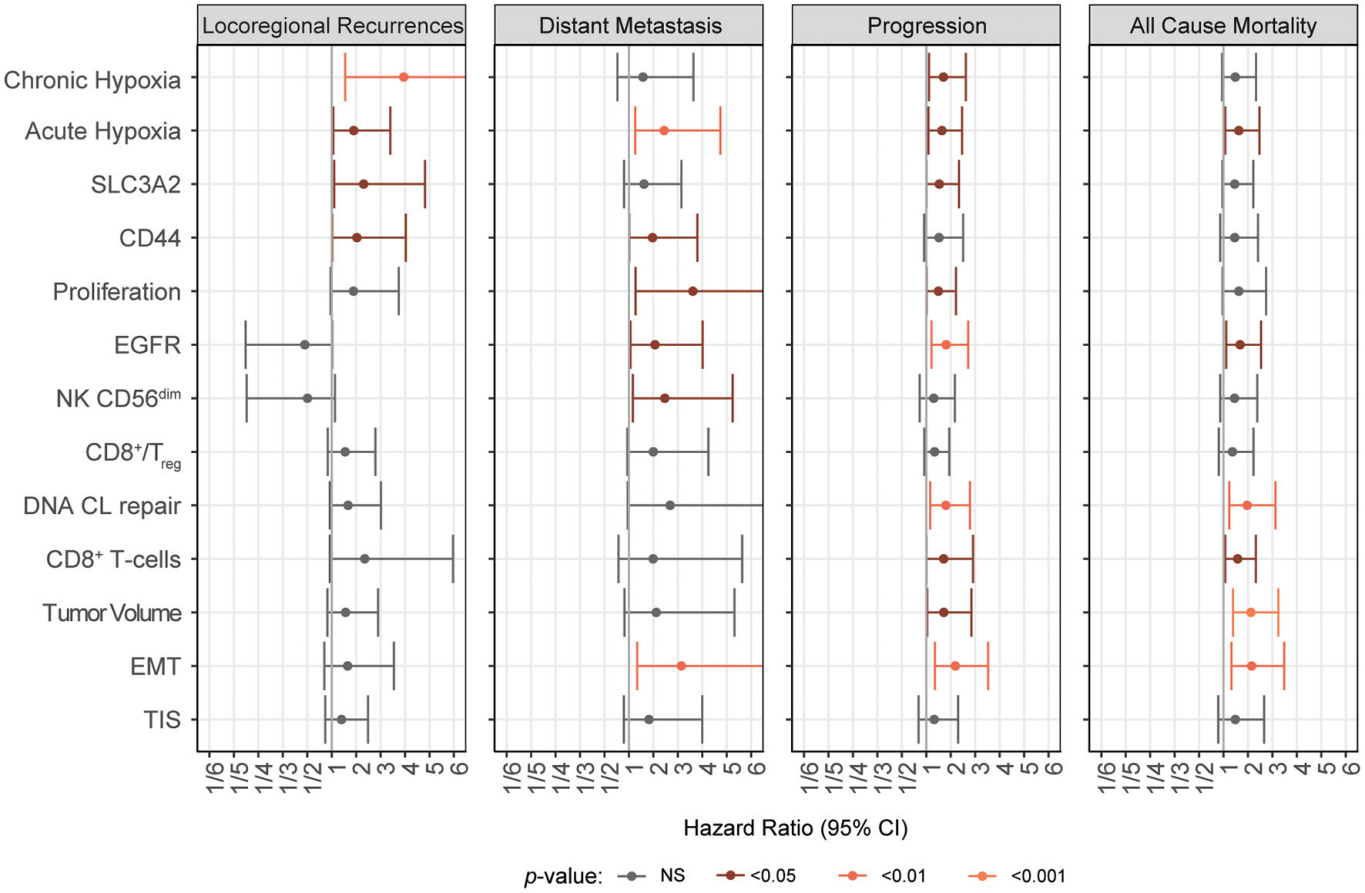
Relevance of biological markers for HNSCC chemo-radiotherapy outcomes. Forest plots comparing individual biological markers across different outcome endpoints are shown. Hazard Ratios for locoregional recurrences, distant metastasis, disease progression and death are from multivariate Cox proportional hazard analyses of the dichotomized cohorts using bootstrap analysis defined cut-offs for all outcomes analyzed in this study. For each marker the “low” score or low expression group was used as reference. Each individual model contained the same clinical variables as those used to determine the cut-off. Only the biological markers are shown.

Cut-offs with the most stable clinical association were selected for each biomarker for further analysis as depicted in Figure 2A and listed in Supplementary Table 2. These analyses confirm that both, chronic and acute hypoxia, are strongly associated with locoregional control. Using these calculated cut-offs in multivariable analyses with clinical factors, we find that among all chronic hypoxia is most strongly associated with a failure of locoregional control (HR = 3.95, *p* = 0.0038) followed by acute hypoxia (HR = 1.9, *p* = 0.03) and stem cell related, SLC3A2 (HR = 2.31, *p* = 0.026) and CD44 (HR = 2.03, *p* = 0.043; Figures 2B, 3; Supplementary Table 3). Although not significantly, larger tumor volumes showed a trend toward worse locoregional control with a hazard ratio (HR = 1.63, *p* = 0.11) that is comparable to those previously reported by others (27). It should be however noted that most tumors in this cohort are relatively large and stage III/IV. This and the fact that the LRC measure also includes regional recurrences, may together affect the specific HR values. Trends toward a worse LRC prognosis were observed in patient groups with tumors with high proliferation and CD8+ T cell scores (HR = 1.89, *p* = 0.067 and HR = 2.35, *p* = 0.071, respectively) (Supplementary Figure 4 and Supplementary Table 3).

To delineate this from general poor prognosis pattern and to investigate the radiation response link further, we repeated these analyses and compared the role of these biomarkers for overall survival, progression free survival and distant metastasis (Figure 3). Cut-off values were defined by the bootstrapping method described above for each biomarker; and multivariable Cox proportional hazard analyses with clinical factors were performed. Notably, gene expression signature scores or expression value cut-offs, as determined by their potential relevance in the 10,000x bootstrapping method, resulted to be different in some of the biomarkers such as “acute hypoxia,” “chronic hypoxia,” “EGFR,” “TIS,” “NK CD56^dim^.” and “CD8+/Treg” (Supplementary Figures 4–6 and Supplementary Table 2).

Most markers show an association with several of the outcome parameters. We find that distant metastasis is associated with EMT (HR = 3.14, *p* = 0.0086), acute hypoxia score (HR = 2.44, *p* = 0.0086), NK CD56^dim^ score (HR = 2.47, *p* = 0.019) and EGFR expression (HR = 2.07, *p* = 0.032) (Supplementary Figure 5, Supplementary Table 3). Poor overall survival is associated with increased tumor volume (HR = 2.12, *p* = 0.00054), EMT score (HR = 2.15, *p* = 0.002), DNA CL repair defect (HR = 1.97, *p* = 0.0043), acute hypoxia (HR = 1.62, *p* = 0.023) and EGFR expression (HR = 1.68, *p* = 0.014) (Supplementary Figure 6, Supplementary Table 3). High EMT (HR = 2.19, *p* = 0.0014), EGFR (HR = 1.82, *p* = 0.0038), acute hypoxia (HR = 1.64, *p* = 0.017), DNA CL repair defect (HR = 1.8, *p* = 0.0085) and chronic hypoxia scores (HR = 1.7, *p* = 0.015) and tumor volumes (HR = 1.72, *p* = 0.036) are associated with a worse progression free survival (PFS) (Supplementary Figure 7; Supplementary Table 3). Interestingly, when comparing the distant metastasis and locoregional control failure data, locoregional control is increased in tumors with higher EGFR expression or containing few CD56^dim^ NK cells while high values in both result in an increased risk of DM. It should be noted, however, that the bootstrapping defined cut-offs were different in both. Yet, as evident from the bootstrapping data chronic hypoxia was not linked to DM but LRC at many cut-offs. On the contrary, the similar shape of the results from the acute hypoxia bootstrapping supports its relevance in both, LRC and DM (Figure 2 and Supplementary Figure 5).

Taken together, for biomarkers which have been previously reported to be prognostic in HNSCC these analyses validate their role in an independent data set. Most biological factors as determined by the selected biomarkers are significantly associated with PFS thereby confirming their relevance. Overall, we find a prominent role for acute and chronic hypoxia and CD44 and SCL3A2 in our cohorts. We show that, from all, chronic hypoxia appears to be the most specific to LRC. In contrast, HR values from EMT and proliferation based splits are greater when assessing DM. Furthermore, these data reveal a role for the immune cell and proliferation related biomarkers in HNSCC outcome after definitive chemo-radiotherapy.

### The Relative Role of Biological Factors in Chemo-Radiotherapy Outcomes in HNSCC

**Figure 4:**
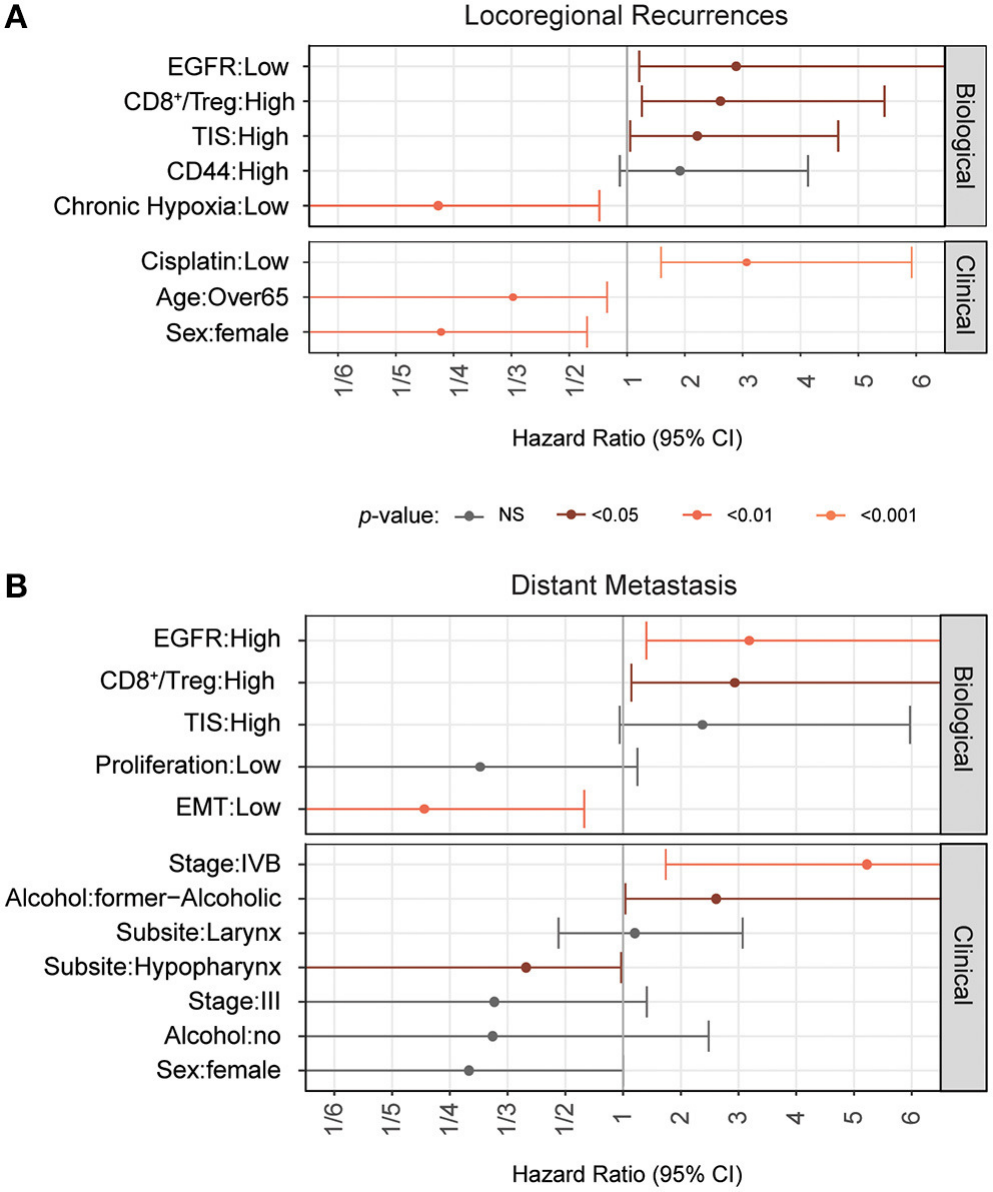
Relative role of biological markers in combined analyses. Forest plots with results from full multivariate Cox Proportional Hazards models are shown. The model was generated using a backward selection procedure. The most frequent level was used as the reference level for this analysis. A Cox proportional hazard model was fit that included all biological markers and clinical variables. Then, each variable was individually eliminated from the model and improvements in model performance were assessed. This process was repeated with the best performing models until the removal of variables did no longer improve the models. Hazard ratios for locoregional recurrences **(A)** and distant metastasis **(B)** as determined by the final model are shown.

The biological markers have been tested independently of each other and most are significantly associated with patient outcome thereby supporting their role in HNSCC and treatment response. However, tumor biology and determinants of radioresistance are multifactorial and may depend on the context and relation to each other. We therefore aimed to identify the most relevant markers in a multivariable analysis. To this end we used a backward selection method. This method creates a Cox proportional hazard model using all available factors. It then iteratively eliminates the least relevant factor until no further decrease in AIC, a measure of model performance, is possible. From these analyses (Supplementary Table 4), we conclude that chronic hypoxia, EGFR expression, CD8+/Treg, T-cell infiltration, and CD44 are the most relevant biological factors that are associated with locoregional control. Multivariable analyses (Figure 4) also demonstrate that they are independent from relevant clinical factors such as cumulative cisplatin dose or sex. Cisplatin dose, age and sex are the clinical factors most associated with locoregional control in this cohort (Figure 4 and Supplementary Table 4). Broadly consistent with the results from the multivariate analyses that were performed on the biomarkers on an individual basis, EGFR and immune cell related factors remain important in instituting an increased risk for distant metastasis, while chronic hypoxia and CD44 are less relevant. Instead, tumor EMT and proliferation affects progression free survival most profoundly and independent of other important factors such as tumor volume or cisplatin dose (Supplementary Figure 8 and Supplementary Table 4). The consistent worse prognosis and distant metastasis association of patients with tumors that score high in the CD8^+^ T cells and related gene expression signatures is remarkable. High CD8^+^ T cell scores, as determined by these signatures or by immunohistochemistry, have been reported to be linked to good prognosis in other heterogeneous HNSCC cohorts (6, 56, 77) and prompted us to analyze this further (Supplementary Figures 9, 10). These analyses suggest that the lack of a good prognosis association could be based on the absence of HPV-positive HNSCC which show overall higher CD8 expression and CD8^+^ T-cell signature scores in the TCGA cohort (Supplementary Figure 9A). Within the HPV-positive group, high CD8 expression is strongly associated with good prognosis (Supplementary Figure 9B). Notably, CD8A/B expression and CD8^+^ T-cell signature values do not correlate well (Supplementary Figure 10). Interestingly, the observed outcome associations in HPV-negative HNSCC appear to be dependent on cumulative cisplatin dose (Supplementary Figure 10).

**Figure 5:**
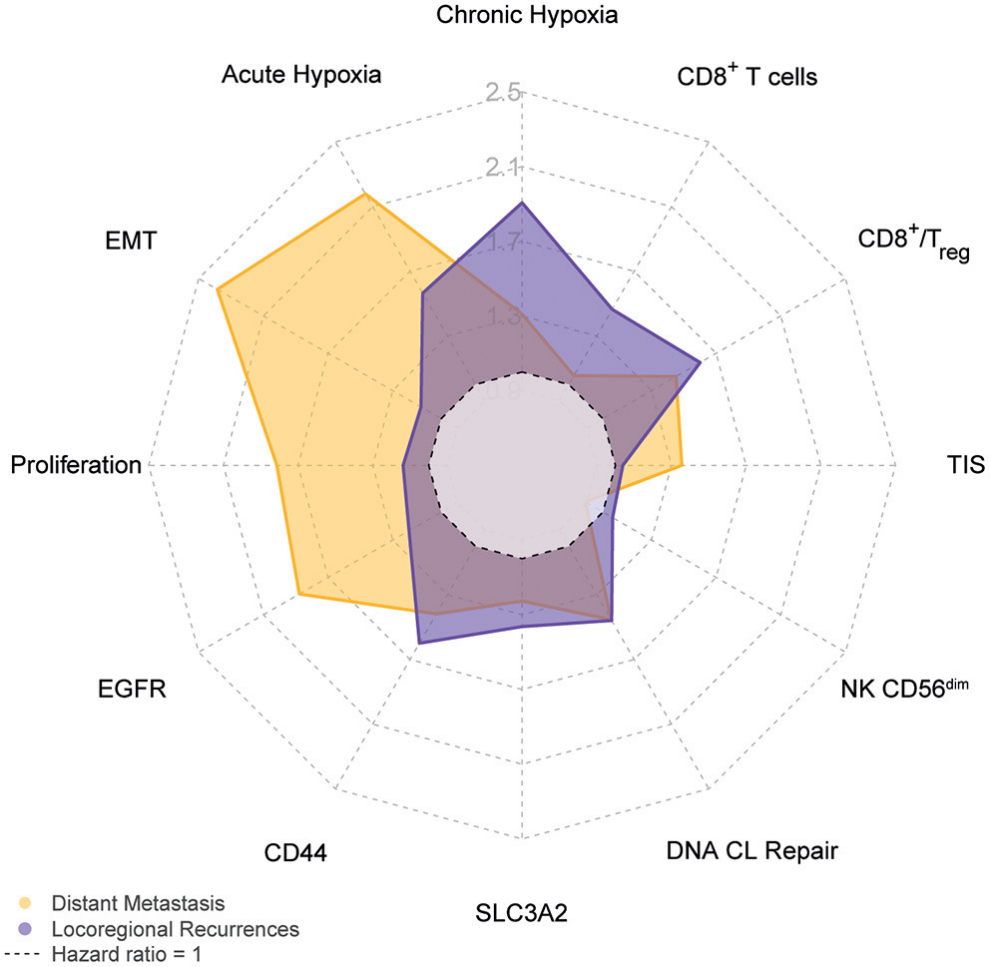
Divergence in biological parameter role for locoregional or distant control. The spider plot depicts the average hazard ratio as obtained after testing all possible cutoffs in a multivariable Cox proportional hazard analysis that included relevant clinical factors as shown in Supplementary Figure 8 and described in Materials and Methods. Hazard ratios for locoregional recurrences are shown in purple and distant metastasis hazard ratios in yellow.

The obvious divergence in the biomarker associations with local treatment outcomes compared to DM development risks prompted us to investigate this further. Unable to classify patients according to “true” biological parameter classes, we relied on bootstrapping methods to provide cut-offs for each outcome endpoints. As described above those resulted to be largely different in some cases such as for EGFR expression and pointed to a different influence in the respective biological mechanisms. We therefore compared the biological markers with respect to their influence in locoregional control or DM risk in a less cutoff-dependent manner by computing the AUC of the hazard ratio plots from multivariable regression analysis with clinical variables (Supplementary Figure 11). Figure 5 shows an overview of the impact of the individual biological parameters on locoregional control or distant metastasis risk. This analysis highlights the difference in the collection of the most relevant survival determinants for each outcome endpoint. Notably, locoregional control is mainly determined by chronic hypoxia, but also acute hypoxia. CD44 expression and CD8^+^ T-cell/Treg ratio are more relevant to LRC than DM, whereas distant metastasis is predominantly influenced by EMT, acute hypoxia, proliferation and EGFR status.

## Discussion

Here, we aimed to evaluate the relevance and interrelation of biological factors known to influence radiation response as determined in preclinical studies. Limited by the size of the study cohort, we restricted the study to markers for which discriminative power has been reported in clinical data in HNSCC and added clinical or biological factors that have shown an important association with radiotherapy outcomes. Using RNA-sequencing data from a large and relative uniform cohort of 197 HPV-negative advanced stage HNSCC patients that were all treated with cisplatin-based chemo-radiotherapy, we find an important role of immune cell (T cell) markers in locoregional control which suggests a role in radiation response. We also show that chronic and acute hypoxia are robustly associated with locoregional control. Similarly, we validated the equally important role of CD44 and SCL3A2, in part related to stem cells, in our study cohort. When assessed in combination, hypoxia, immune cells, EGFR are the most discriminating independent factors in LRC. For DM, those also include EMT. Overall, considered in the context of clinical factors and each other, our study underscores the relevance of many of these biological factors in HNSCC chemo-radiotherapy outcomes.

To advance previous findings on determinants of chemo-radiotherapy outcomes and to prioritize prognostic markers for multi-parametric prediction models, we focused on (i) the validation of expression-based prognostic markers in the chemo-radiotherapy setting and (ii) the evaluation of their complementarity and (iii) the assessment of any dependence to important clinical or other biologic factors. Many HNSCC studies highlight the role of biology for outcome (3–6, 78). A major drawback however for many of such studies is the heterogeneity of the HNSCC patient cohorts or a lack of contextual analyses (78). If focused on tumor site they often encompass many different treatments or if focused on treatment they combine different tumor sites, HPV-negative and positive. Large multicentric studies are therefore valuable contributions to the field (12, 13, 15, 16, 57) that provide insights to the biology of HNSCC and its link to patient outcome (5, 6, 27, 28, 79, 80). Clinical factors are important (81, 82) but are often not considered in multivariable analyses (78, 83). A lack of tumor volume data for example, even though clearly linked to LRC (81, 84–86), impedes the assessment of a role for or a bias from tumor volumes in such analyses. To minimize such treatment or tumor site related bias due to possible interactions; we deliberately excluded the biologically distinct oral cavity and HPV-positive oropharyngeal HNSCC (14, 16, 87–90) in our study. Our cohort also solely comprises definitive chemo-radiotherapy-treated advanced HNSCC. In contrast to predetermined gene sets in nanostring technologies, the availability of full transcriptomic data by RNA-Seq allowed us to test selected gene expressions and signatures related to the biological processes that we queried. Together we were able to show that most of the selected markers or marker categories are not related, are independently linked to outcome and that outcome associations are not based on links with known important clinical factors. Overall, we observed little or no influence or interactions with clinical factors, with the notable exception of tumor volume and cumulative cisplatin dose, factors often not accounted for in other biomarker studies. While correlations within the immune markers were expected, here we reveal an association with acute hypoxia scores which in turn appears to be linked to proliferation. Such relations or complementarities can alter the prognostic value or impede a discrimination of the true source of the observed outcome relevance. It however highlights the importance to study such markers in the context of each other and within the same cohort. Overall, our study pinpoints expression markers that should be considered as valuable contributors of future multi-parametric prediction models that combine clinical, radiologic, pathological and genetic variables for improved prognosis in advanced HPV-negative HNSCC (91, 92).It is difficult to discern factors that determine tumor radioresistance (83). A comparison of similar patient cohorts treated without or with different doses of radiotherapy would be required to strengthen such a link. Since cisplatin-based chemo-radiotherapy has become a standard treatment for HNSCC to improve quality of life by achieving organ preservation, surgically resected HNSCC patients with similar clinical tumor characteristics are rare, impeding such comparisons. However, in the absence of a comparable but non-radiotherapy treated study cohort, differences in LRC (mostly achieved by radiotherapy) as defined by the biomarker classification, can suggest a role in radiation response. In our study, we assured that important clinical factors that impact patient outcomes have been considered to limit bias or dependence. DM events may have occurred prior to LRC events and could have masked a greater impact in radiation response, such as in the case of acute hypoxia that also shows a strong association with DM. The comparison of LRC with DM further helped to discern a more radiation response specific role from a role in metastasis. Our data here and those reported by us and others do indeed confirm the role of hypoxia in determining radiation response as reflected by the LRC rates (25, 27, 29). In addition, hypoxia has been also implicated in tumor cell invasiveness, facilitating dissemination, and has been therefore associated with metastasis formation, a role that is also evident from our DM analyses. Similarly, we have recently shown that HNSCC cell lines with DNA crosslink repair defects are more migratory and invasive (63), a feature that may explain the association with DM prognosis but could also result in a greater regional spread and failure of locoregional control.

After the initial EGFR studies in clinic and the success of cetuximab combinations (40, 93, 94), cetuximab in HNSCC and the role of EGFR amplification and expression have been disputed since then (95). Most of these studies focused on the very high expressing or used a median cut-off to detect an association with the clinical endpoints analyzed. Here we see a clear role for EGFR in the outcome data when also considering hypoxia and other factors in multivariable analyses. Average to high EGFR expression, is linked to improved LRC when analyzed individually. The association of a low EGFR expressing group with poor LRC however becomes much clearer in the combined multivariable analyses that integrated all relevant biomarkers. It epitomizes the importance of combined analysis, as the prevalence of other, also clinical, factors in the different EGFR expression classified groups may have shifted or masked a possible influence in other studies if not accounted for, as revealed here. Given its role in promoting cell cycle progression, it is conceivable that increased EGFR levels mediate an increase in tumor repopulation between fractions; a radiotherapy response determining process that is counteracted by radiotherapy treatment acceleration or concurrent chemotherapy. This process is therefore limited in our patient population in contrast to some earlier studies that analyzed the influence of EGFR (96). Notably, the association with improved LRC is still discernible (HR = 0.57, *p* = 0.067) when reanalyzing the data using the higher EGFR expression cut-off that was used for the DM data. DM HR values however drop to 0.57 (*p* = 0.2) showing a DM link only in the top 25% EGFR expression group (HR = 3.19, *p* = 0.0056). This more aggressive nature of highly EGFR expressing tumors is consistent with other reports in HNSCC and other cancer types (97).

Our study is limited by statistical constraints due to the cohort size. This enforced us to limit the biological variables and apply selection processes such as the bootstrapping analyses. Yet, it becomes evident that the prognostic value of many of the factors could be validated in our cohort and withstood multivariable analyses with the important clinical variables. Among the clinical variables, we observe a trend toward poor outcomes in current smokers, however this does not reach significance in our cohort. Low numbers in the former smoker category but also the lack of more accurate smoking status values may have decreased the power to reveal the reported association with smoking (79, 98, 99). Since we focused on known determinants of radiation response, other biomarkers were not included despite their relevance or prognostic value in HNSCC (5, 6, 16, 100–106). Some, such as tumor mutational burden (TMB) are prevalent in laryngeal and HPV-negative pharyngeal HNSCC (14) but require DNA sequencing data. TMB was found to be associated with poor prognosis in HPV-negative chemo-radiotherapy treated patients in our previous study (36) and more strongly so in a cohort of patients that also included oral cavity cancers and HPV-positive oropharyngeal (107). Interestingly, low immune cell infiltration or CD8+ T cell values, as assessed by gene expression, have been assigned to HNSCC high in TMB or mutational signatures related to smoking (56, 107).

Other limitations result from technical challenges. Here we detect different biological processes and factors in clinical samples by using published and validated expression signatures—that are linked to these processes. These gene expression signatures may not be perfect identification tools for the specific biology in question (83); however they often reflect the abundance of certain biological elements well (108, 109). The DNA CL repair defect prediction model has for example been generated using functional endpoints and then validated in independent cell line panels or by genetic modification. On the other hand, markers such as CD44 are less clear defined. CD44 expression is associated with stem cell-ness in tumor cells (110), but it is also expressed under hypoxic conditions or in epithelial cells and is a marker for effector memory T cells (45). Therefore, it is particularly interesting to observe the correlation with SLC3A2 another stem cell related marker in our samples which confirms its link to tumor stem cell abundance. Notably, we find a correlation between acute hypoxia and TIS or CD8^+^ T-cell scores, suggesting a higher T-cell content in acute hypoxic areas or tumors which could be proposed to be driven by hypoxia induced inflammatory cytokine release (111). This T-cell/acute hypoxia correlation may in part also be responsible for the consistent poor outcome association of the CD8^+^ T-cell gene expression signatures. Reiterating the role of technical limitations, it should be noted that these gene expression signatures were based on transcriptional profiles of purified immune cell subsets. Through multiple adaptation steps, they evolved to markers that allowed further discrimination in the context of colorectal carcinoma and HNSCC (56, 75, 76). In terms of identification accuracy there are potential challenges with such technical approaches that can also explain discrepancies with immunohistochemistry determined factors. It is evident that the tumor context affects gene expression of the immune cells and, on the other hand, tumor gene expression features, if present in these signatures, can compound the identification. For instance, the CD8^+^ T cell signature includes ZEB1 expression, a protein involved in EMT and a poor prognostic factor in HNSCC (56, 76, 112–115). We therefore assessed CD8A and B gene expression in our samples as a simple surrogate for CD8^+^ T cells and show its limited complementarity with the CD8^+^ T cell signature score and associations with outcome. The better LRC outcome of patients with CD8 positive tumors in the low cumulative cisplatin patient category is in line with previous report based on IHC (116) The lack of an association with outcome in patients that received high cisplatin doses however demonstrates treatment dependence and explains the discrepancy to other studies (6, 77) when considering this clinical variable in our cisplatin treated cohort. Despite a significant but weak correlation with CD8A expression, high CD8^+^ T cell signature values are associated with poor outcomes, demonstrating the influence of the other features in this discriminating signature. Immune cell identification by gene expression may not be flawless. Yet, together, our data indicate a prognosis association that is linked to this particular patient treatment. One could speculate that hematologic toxicities associated with cisplatin administration could contribute to this pattern by abolishing the benefit from an immune cell rich tumor status in these individuals. On the other hand, recent studies suggest an enhancement of antitumor immunity by cisplatin that could also diminish the impact of the pre-treatment immune status (117, 118). While the primary emphasis for prognostic biomarkers lays in the discriminatory power to predict patient outcome, the focus of biomarkers for targeting opportunities is the achievement of an accurate representation of the marked biological process or elements. The signatures used here were selected based on their reported association with both immune cell infiltration and prognosis in HNSCC (56, 76). Yet the question remains whether they reflect CD8^+^ T cell infiltration well.

Interestingly, we did find a seemingly independent and consistent role for CD8^+^, non-regulatory, T cells in our study cohort. Observed for resected HNSCC in overall survival outcome data before, here we show an association with both, LRC and DM, in chemo-radiotherapy treated HNSCC patients indicating those with a high abundance of such T cells to have a worse prognosis. To our knowledge this poor prognosis association with radiation response has not been reported previously (6, 77, 119). As detailed above, this discrepancy with other studies is only in part explained by the used technology (116, 120) (IHC CD8 expression vs. gene expression signatures) since the signatures showed a good prognosis association in the Mandal et al. (56). Careful inspection of the TCGA data revealed increased CD8^+^ T-cell gene expression signature scores in the HPV-positive oropharyngeal that drive the good prognosis association. A pattern observed in other studies as well (56, 77, 121–124). Mandal et al. adjusted for HPV-associated outcome differences, which does not account for a possible interaction between the two variables (56). The CD8A and B expression HR plots in our analyses however suggest a stronger effect in the HPV-positive subgroup. Despite obvious evaluation challenges when using the different techniques and associated cut-offs, a similar argument applies to other studies based on immunohistochemistry determined CD8^+^ T cell infiltration values. A significant HPV status association got lost in multivariable analyses that indicated a good prognosis association of CD8^+^ T cells in oropharyngeal squamous cell carcinoma patients (59). Yet, some studies also show a good prognosis association with TIS or CD8^+^ T cells in HPV-negative patients using other scorings, cut offs and expression signatures (125). Since the effect size can be small, patient treatment associations with survival are often not significant in small studies. Treatment could however alter prognosis in subsets of patients. For instance, patients with tumors with DNA crosslink repair defects benefit most from a high cumulative cisplatin dose (63). Similarly, possible immune cell infiltration links could depend on treatment. Despite worse PFS in cases that lack or show minimal CD8A or CD8B expression, we cannot observe the previously reported poor prognosis link in CD8^+^ T cell signature low patients in our cohort. No associations between TIS or CD8^+^ scores and clinical variables were found; and outcome association links derived from the correlation with acute hypoxia should have been accounted for by the multivariable analyses. Together, our data suggest a role for HNSCC treatment, in particular cisplatin, in immune cell infiltration determined outcomes. Early cancer immunotherapy trials in HNSCC with immune checkpoint inhibitors demonstrate a benefit and underscore the potential value of immune response and chemo-radiotherapy relevant biomarkers to identify patients that will benefit from such treatments (24, 126–134). Larger comparative studies are therefore needed to disentangle the role of CD8^+^ T cells in the individual genetic HNSCC context and the important clinical variables connected to its role in patient outcome (78, 83, 135).

Our patient cohort is fairly unique in that it consists of definitive chemoradiotherapy treated advanced HNSCC patients. Based exclusively on resected HNSCC, these cases are unfortunately not present in the TCGA data. Supported by the detailed clinical data and follow up, this allowed us to elaborate on the role of biological determinants of chemoradiotherapy response. A quarter of the patients suffered from loco regional recurrences after treatment; a treatment success rate that further stresses the relevance of the biological factors found to determine treatment failure. This study does not provide or test clinically applicable prognostic markers. It was designed to compare the individual factors in relation to each other to assess and understand their influence in HNSCC outcome. Optimal cutoffs identified by the bootstrapping method and illustrated in the hazard ratio plots require validation for further development into true prognostic markers. Based on our results, future studies should focus on the elaboration of prognostic models that incorporate these biological markers together with important clinical factors. The multivariable outcome association results and the lack of correlations suggest that these future models should include all biological factors. Discrepancy in the optimal cutoff values further points to the value of non-dichotomized variables in such efforts and also reveals a possible cause of incongruent outcome associations in previous studies. The value of the clinical factors is exemplified by the fact that some biological markers (i.e., DNA CL repair or CD8^+^ T cells) lose their strength in patients groups with a high cumulative cisplatin dose.

While tumor stem cell targeting agents are still under development, some of the other biological factors are targetable. Next to high-dose alkylating agents, PARP inhibitors may help to exploit DNA CL repair defects (62) and different immunotherapy options are currently being tested in the HNSCC setting (136). The value of such biological markers in personalized treatments remains to be determined; however our study demonstrates that those patients are in need of improved therapy options.

In conclusion, this multicentric external validation study confirms the important and independent role of biological factors that embody hypoxia, stem cell-ness, tumor growth, EMT and DNA repair for locoregional control in chemoradiotherapy treated patients. The multifactorial analyses results highlight the need to consider these biomarkers in the context of each other and also revealed an important role for immune cell abundance in HNSCC treatment outcome.

## Data Availability Statement

The data in this study is available the European Genome-phenome Archive (https://www.ebi.ac.uk/ega/) with the accession numbers: EGAD00001005721, EGAD00001005715, EGAD00001005716, EGAD00001005717, and study number EGAS00001004090.

## Ethics Statement

The studies involving human participants were reviewed and approved by Institutional Review Board The Netherlands Cancer Institute, Amsterdam, The Netherlands; Institutional Review Board VuMC, Amsterdam, The Netherlands; Institutional Review Board Maastro, Maastricht, The Netherlands. The patients/participants provided their written informed consent to participate in this study.

## Author Contributions

CVen, MH, PE, and MJ contributed to the conception and design of the study. MH, RR, CVer, OH-V, FH, PL, HB, CL, MV, RB, and MB contributed to the acquisition of data. MH, RR, and FH established and/or curated databases. PE, MH, and SS performed data analysis. MH, CVen, PE, and MJ contributed to data interpretation. CVen, MH, and PE wrote the first draft of the manuscript. All authors contributed to manuscript revisions and approved the submitted version.

### Conflict of Interest

The authors declare that the research was conducted in the absence of any commercial or financial relationships that could be construed as a potential conflict of interest.
