# Supplementary figures and images for: Biological Determinants of Chemo-Radiotherapy Response in HPV-Negative Head and Neck Cancer: A Multicentric External Validation
Source: Front Oncol. 2020 Jan 10;9:1470. doi: 10.3389/fonc.2019.01470 (PMC6966332; doi:10.3389/fonc.2019.01470)

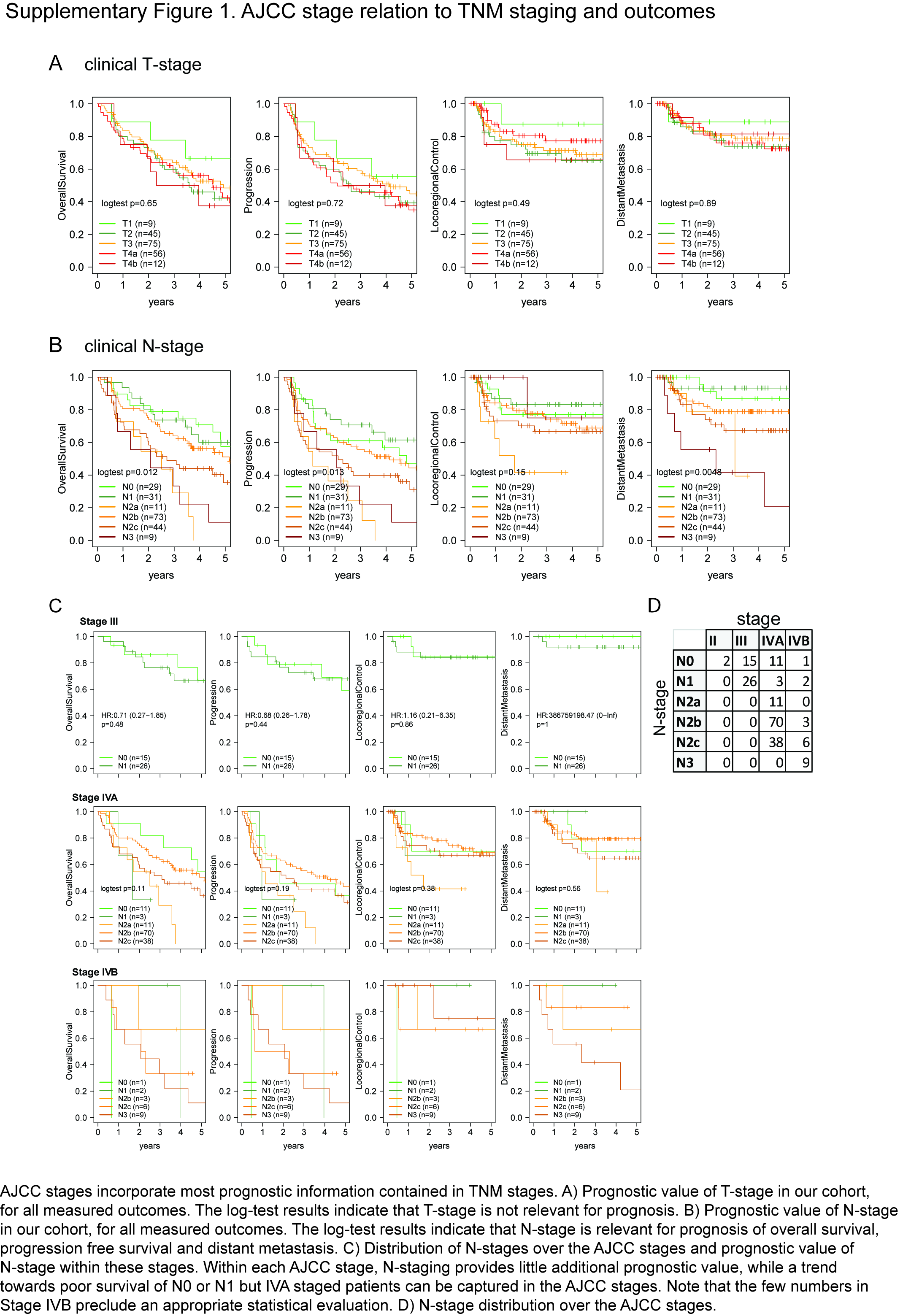

Supplement: Supplementary file 1 [file Image_1.TIF]

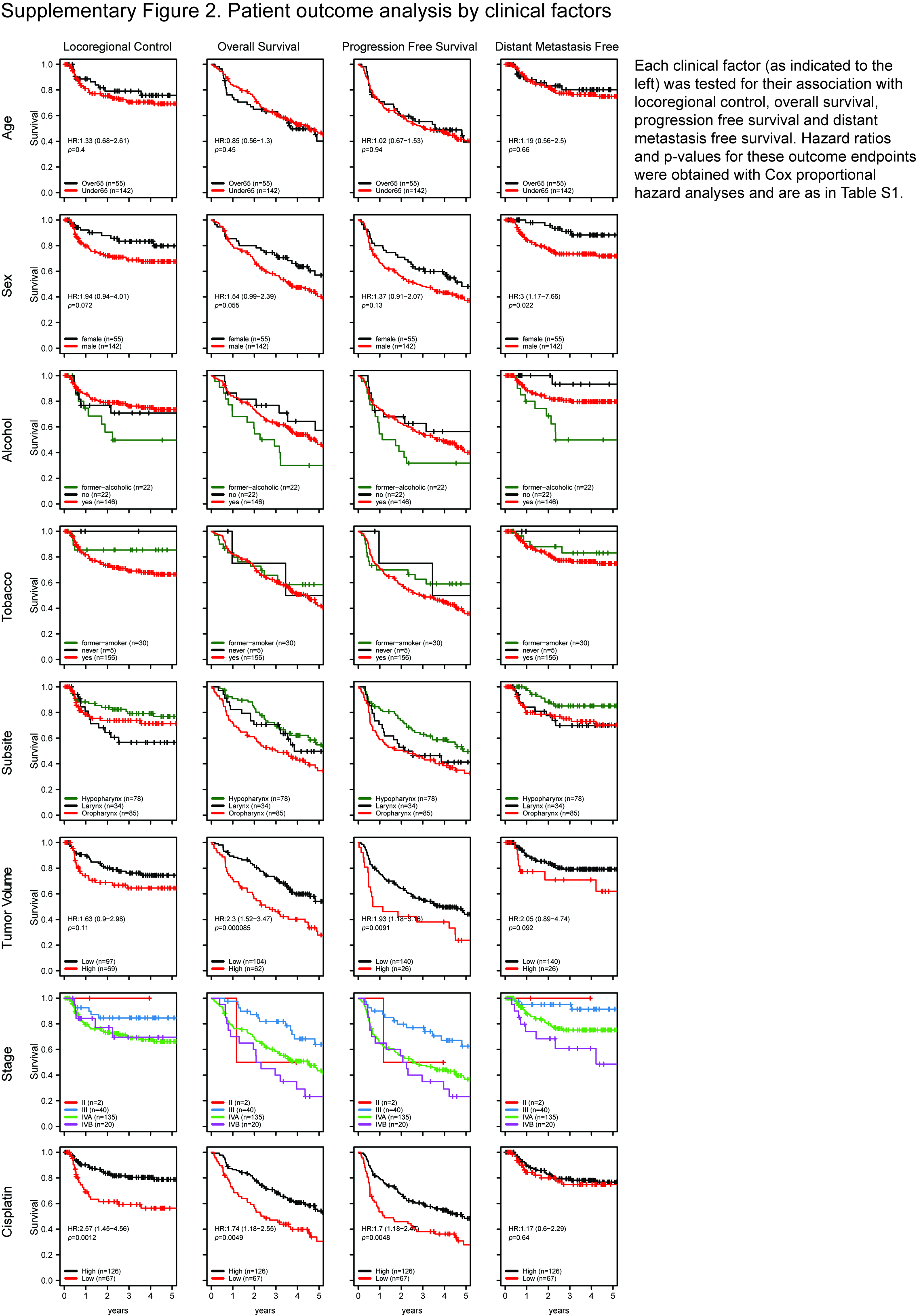

Supplement: Supplementary file 2 [file Image_2.TIF]

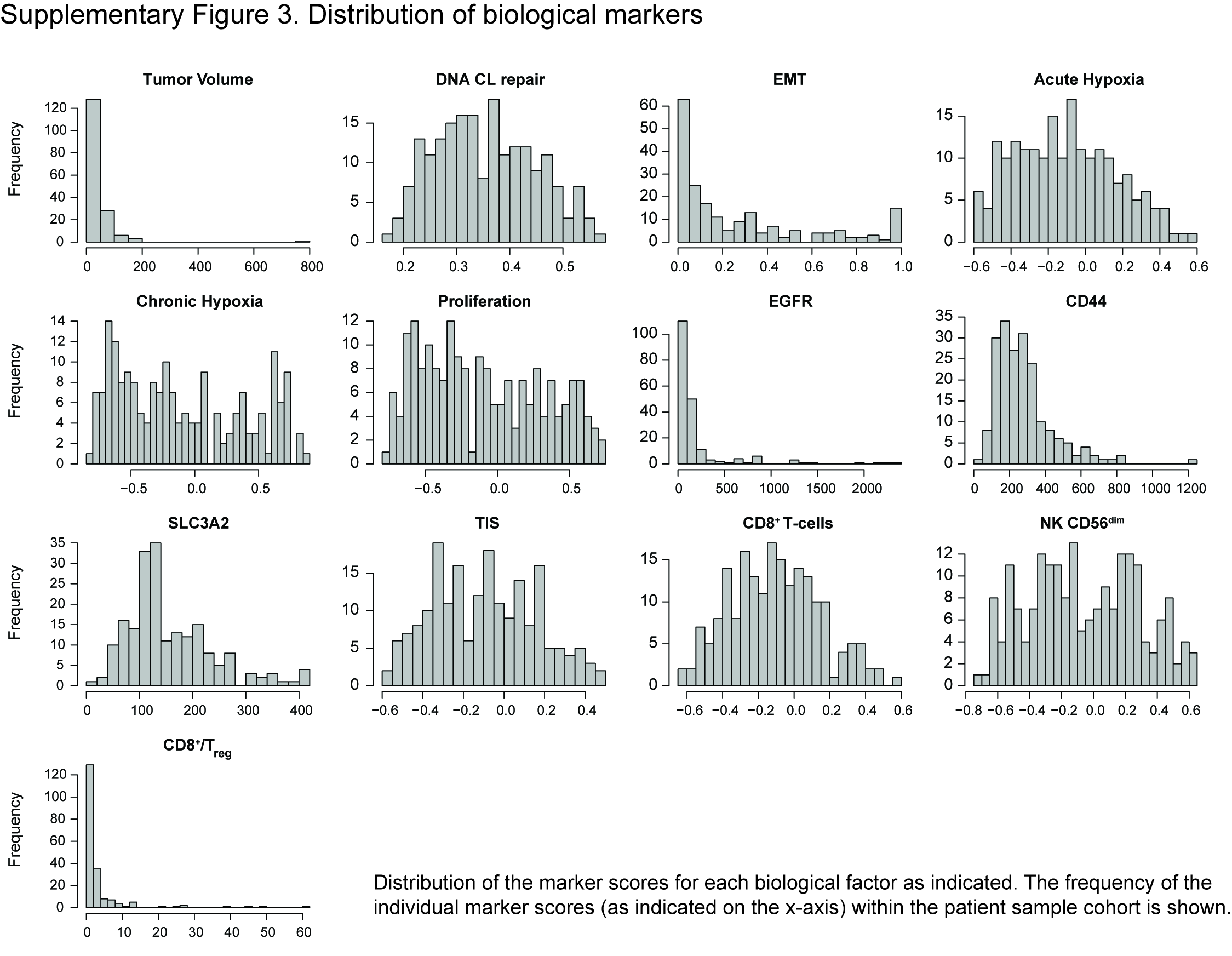

Supplement: Supplementary file 3 [file Image_3.TIF]

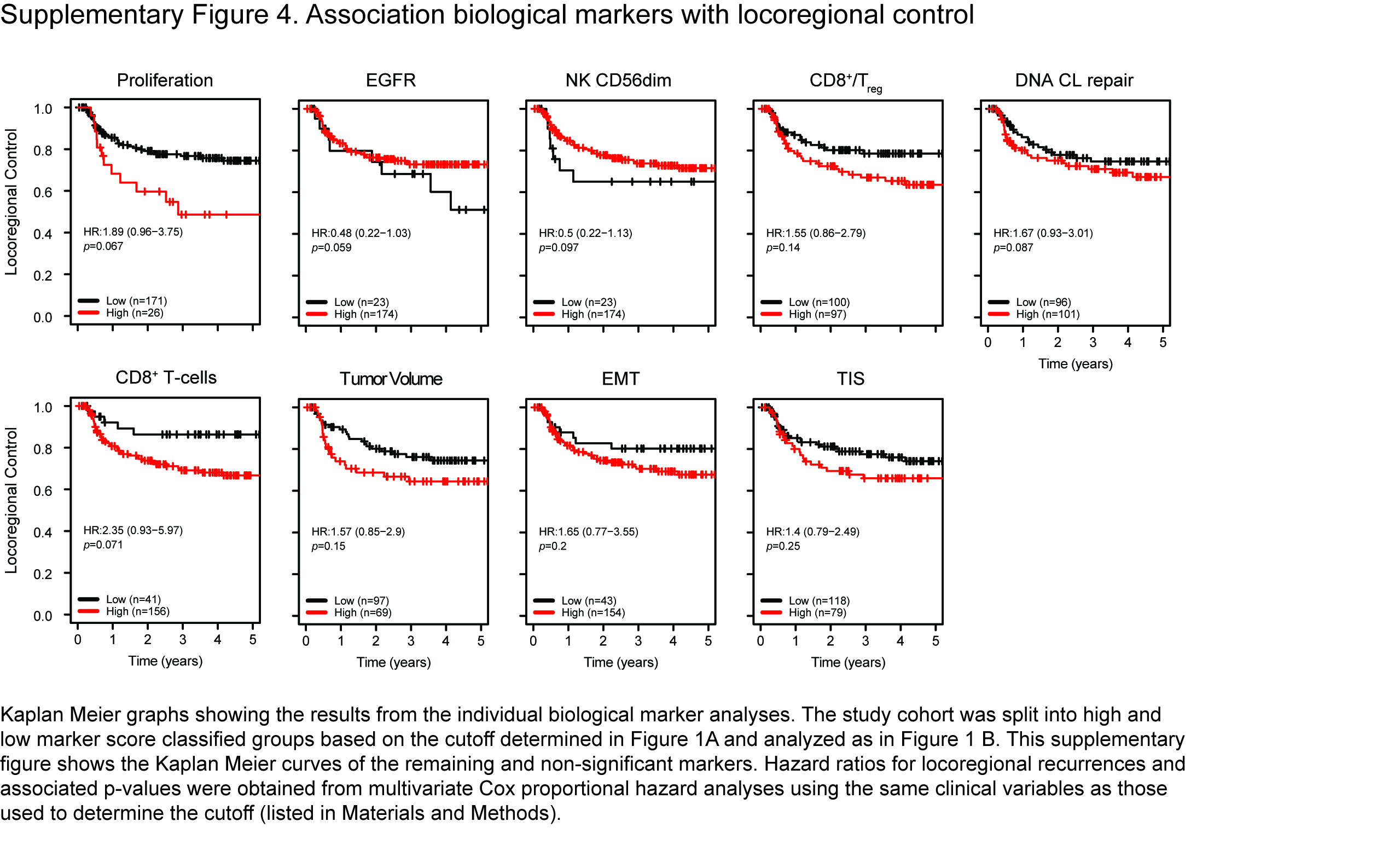

Supplement: Supplementary file 4 [file Image_4.TIF]

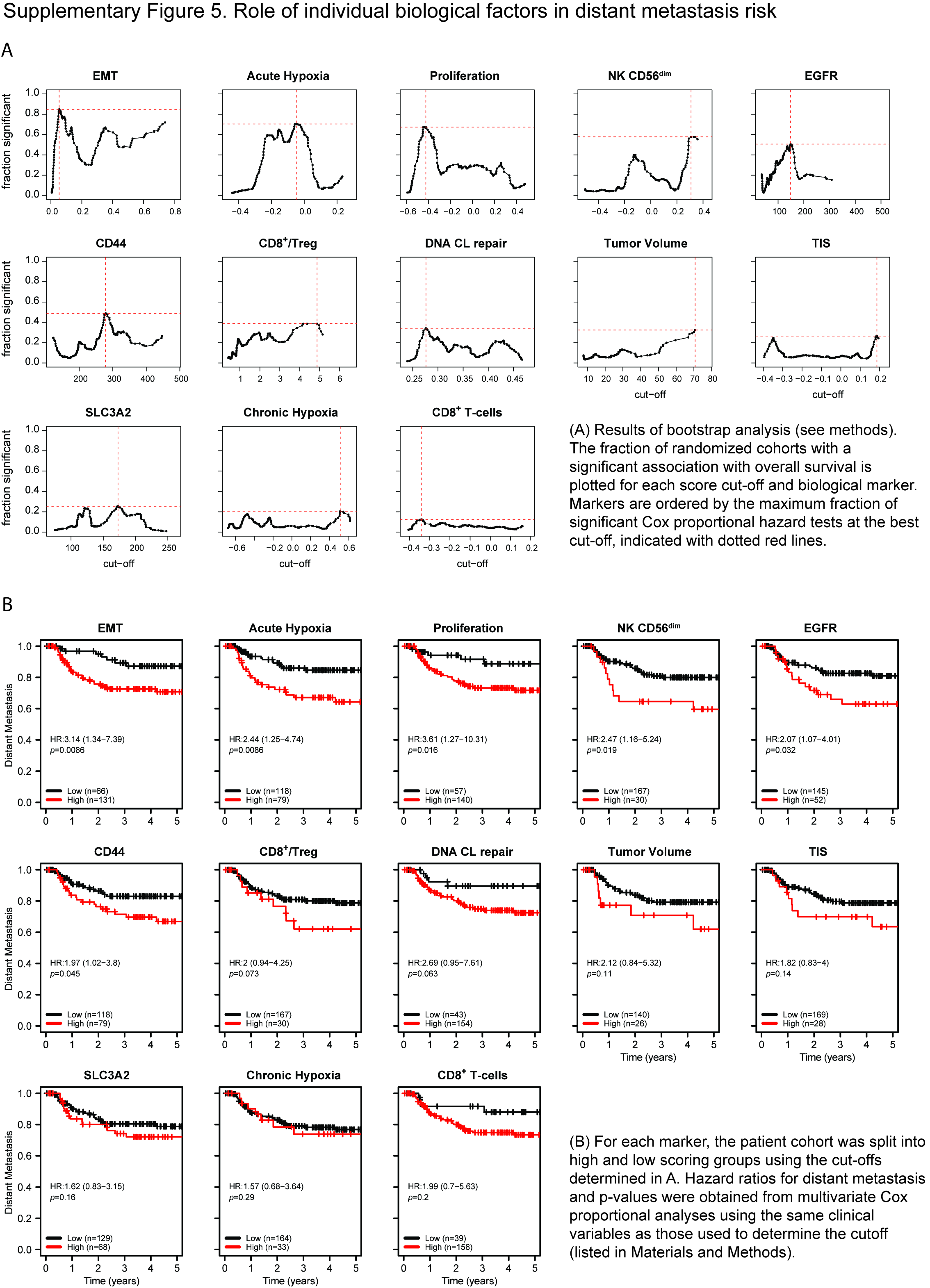

Supplement: Supplementary file 5 [file Image_5.TIF]

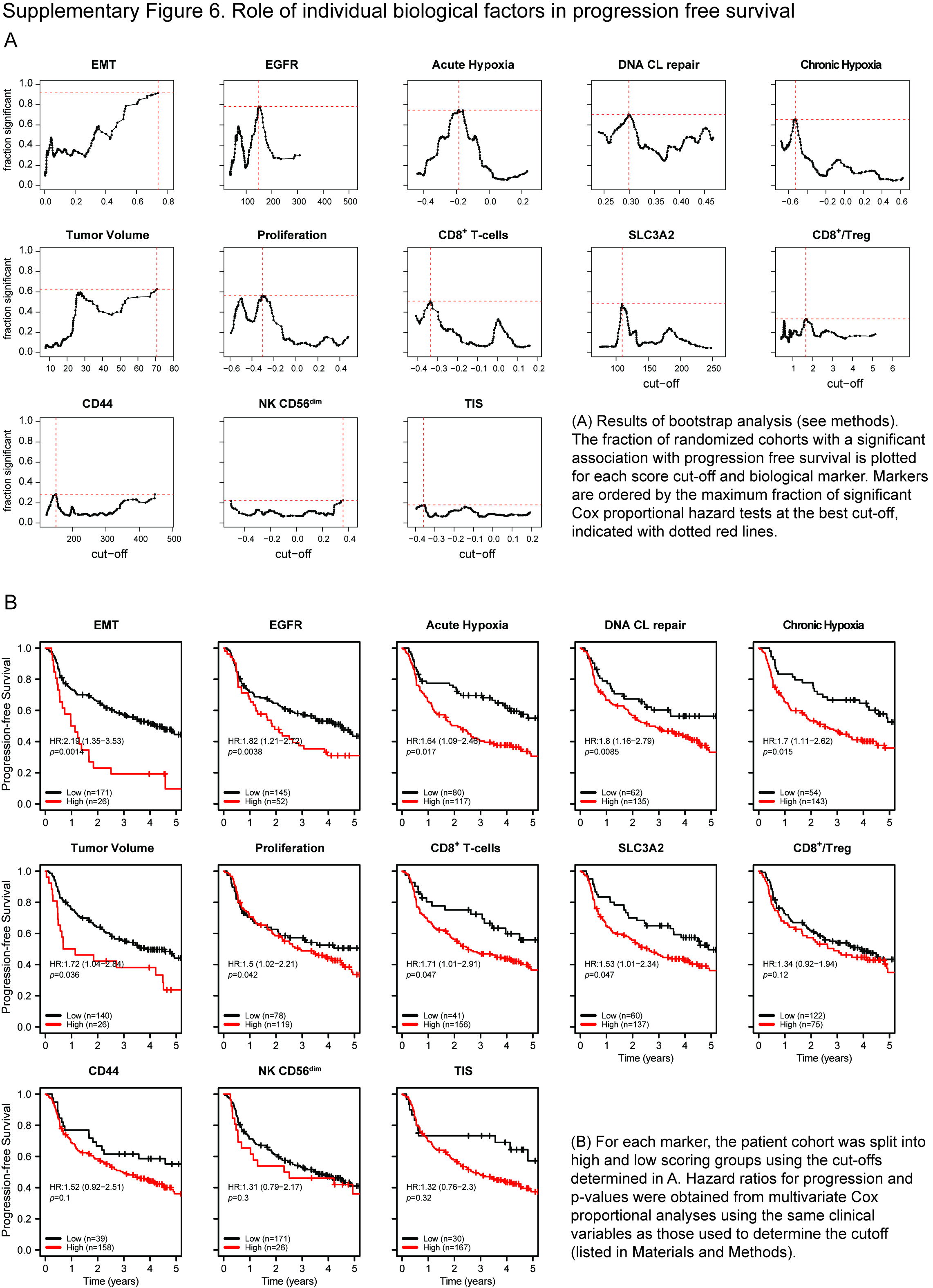

Supplement: Supplementary file 6 [file Image_6.TIF]

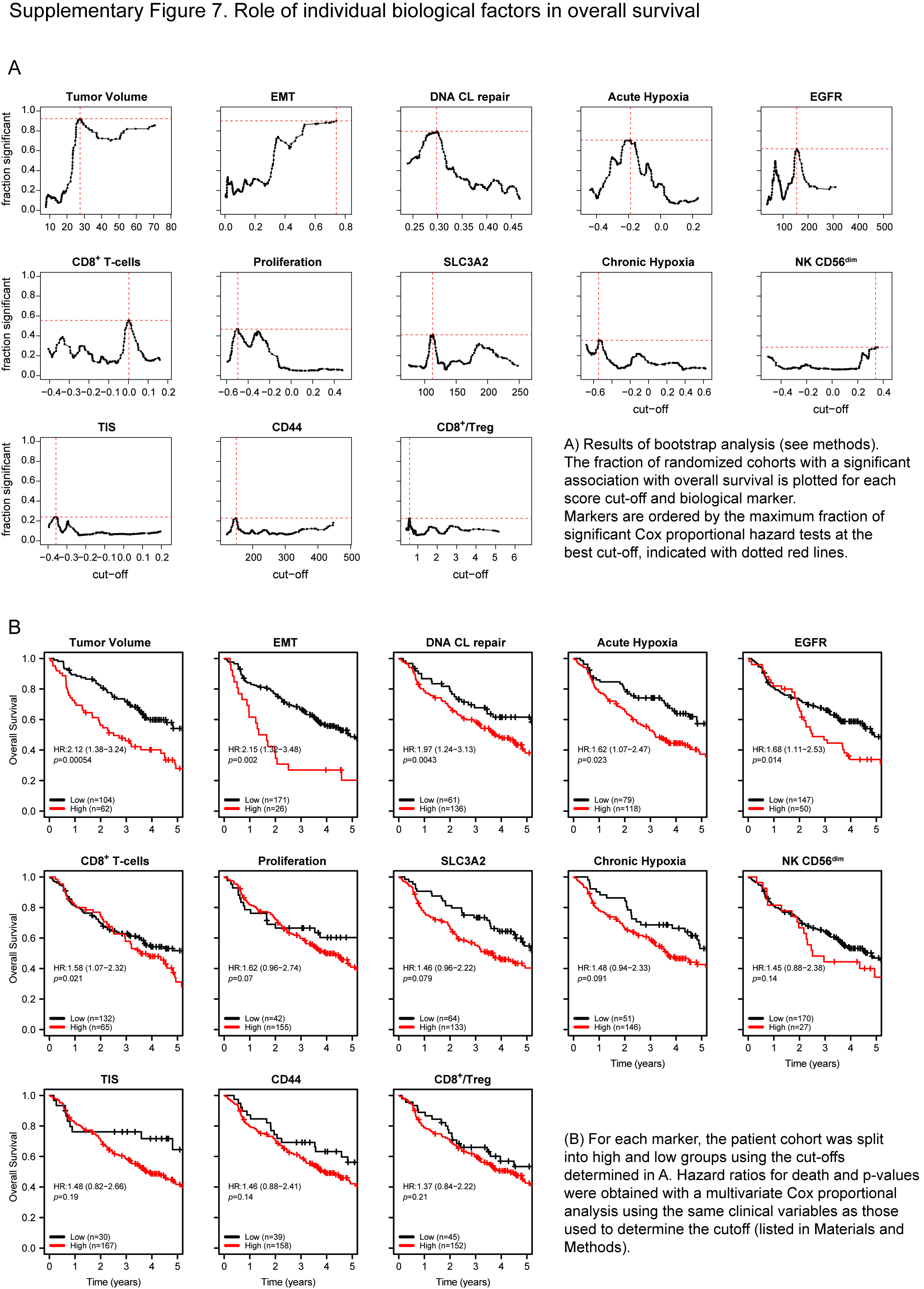

Supplement: Supplementary file 7 [file Image_7.TIF]

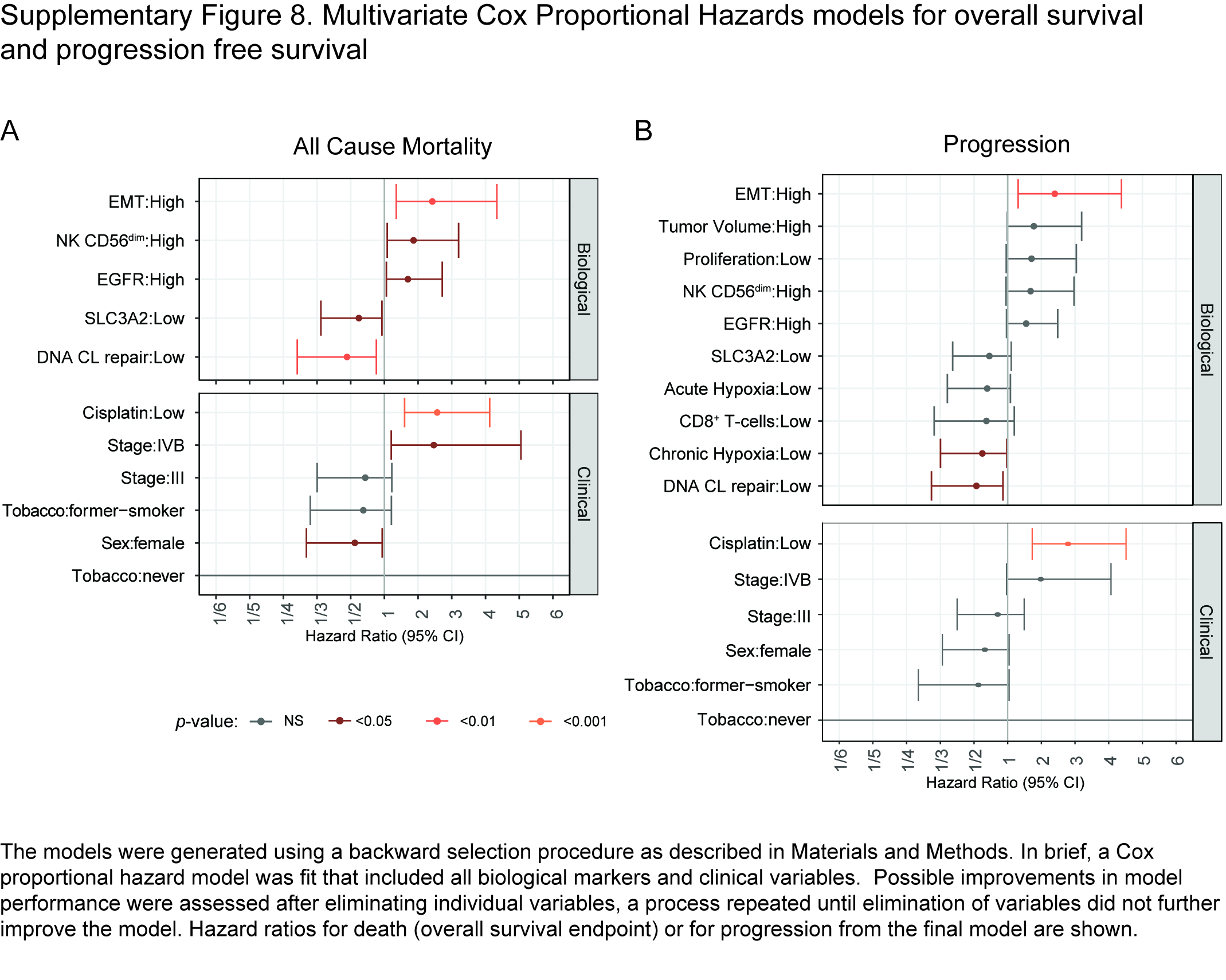

Supplement: Supplementary file 8 [file Image_8.TIF]

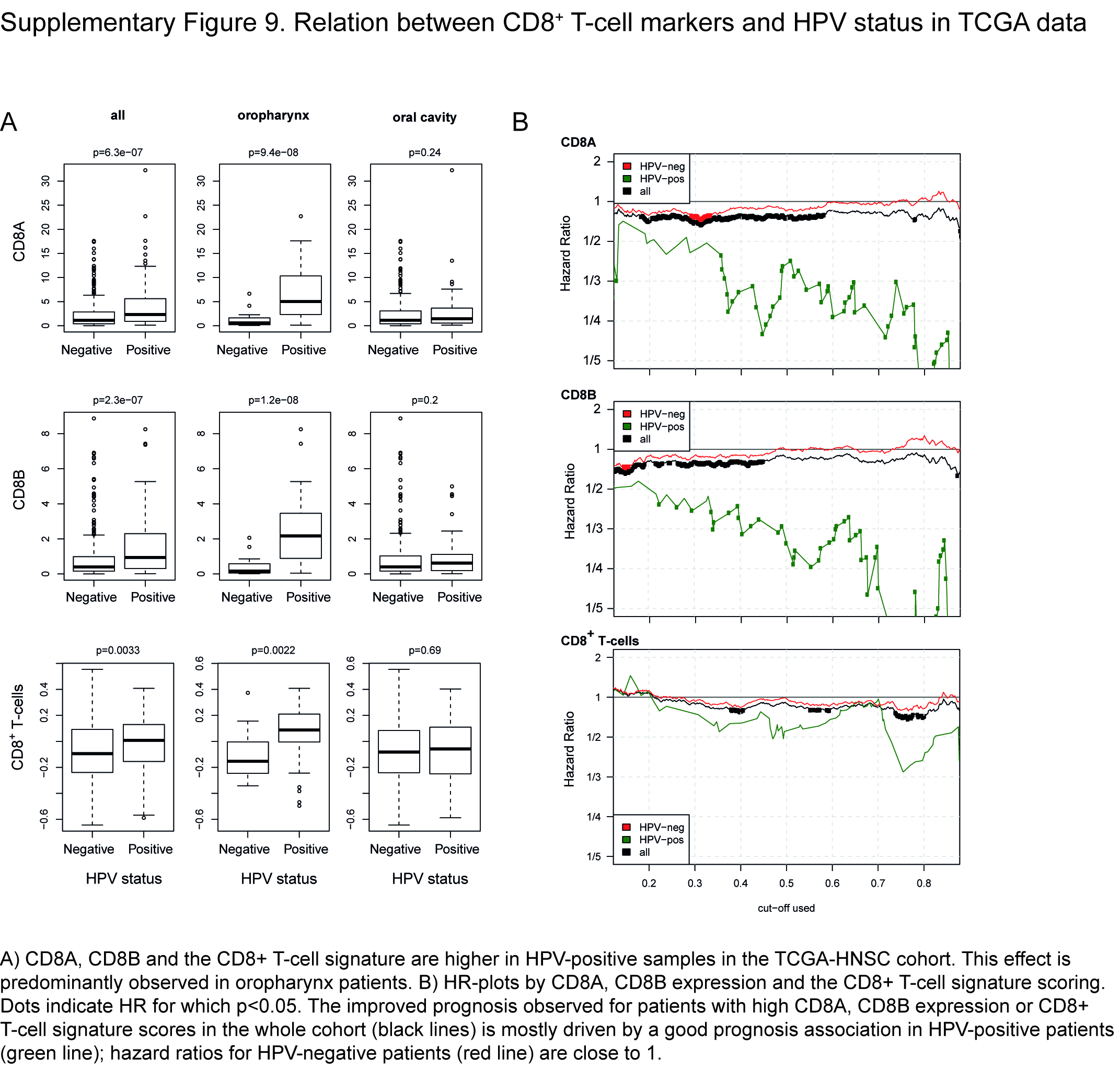

Supplement: Supplementary file 9 [file Image_9.TIF]

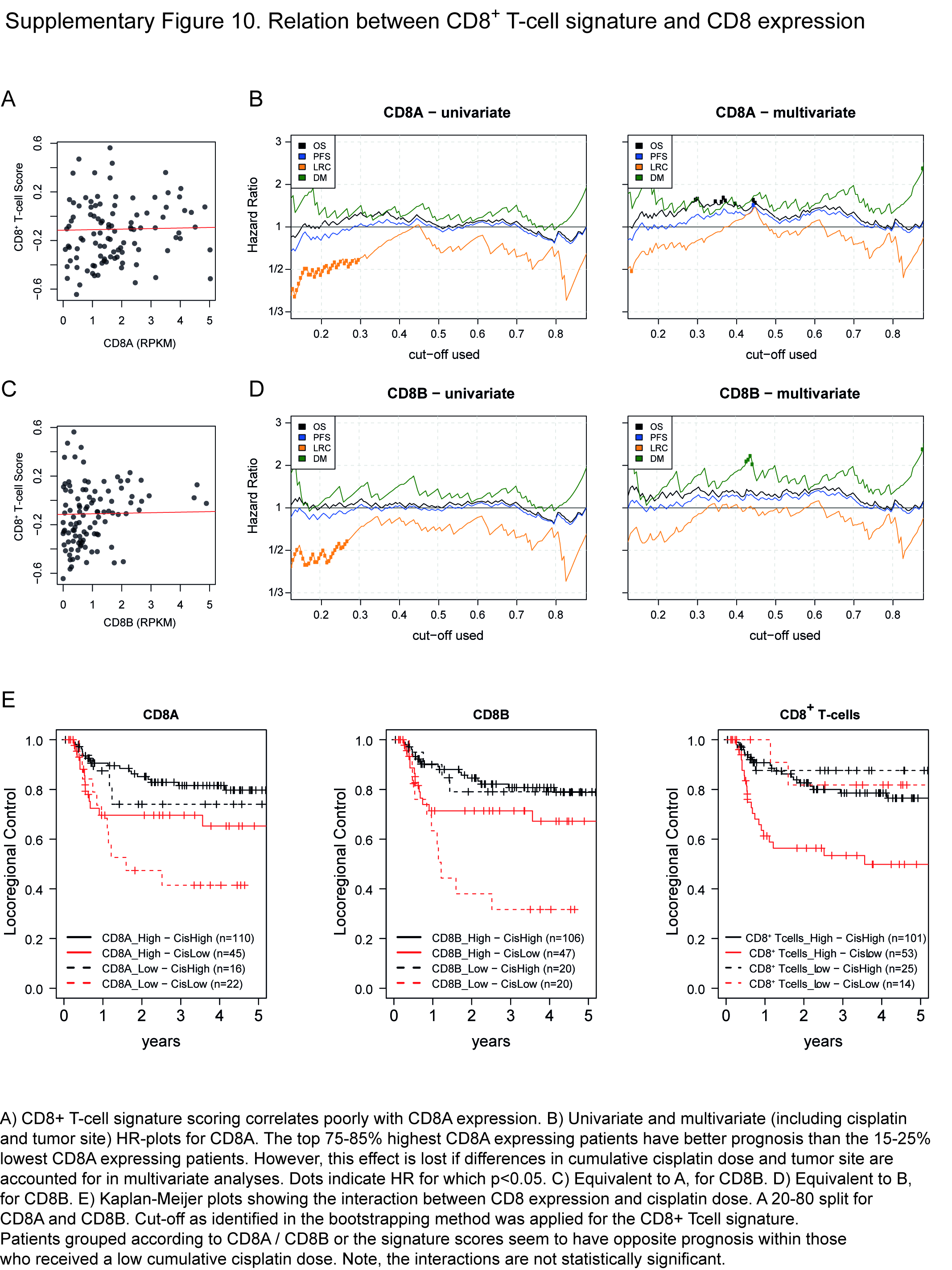

Supplement: Supplementary file 10 [file Image_10.tif]

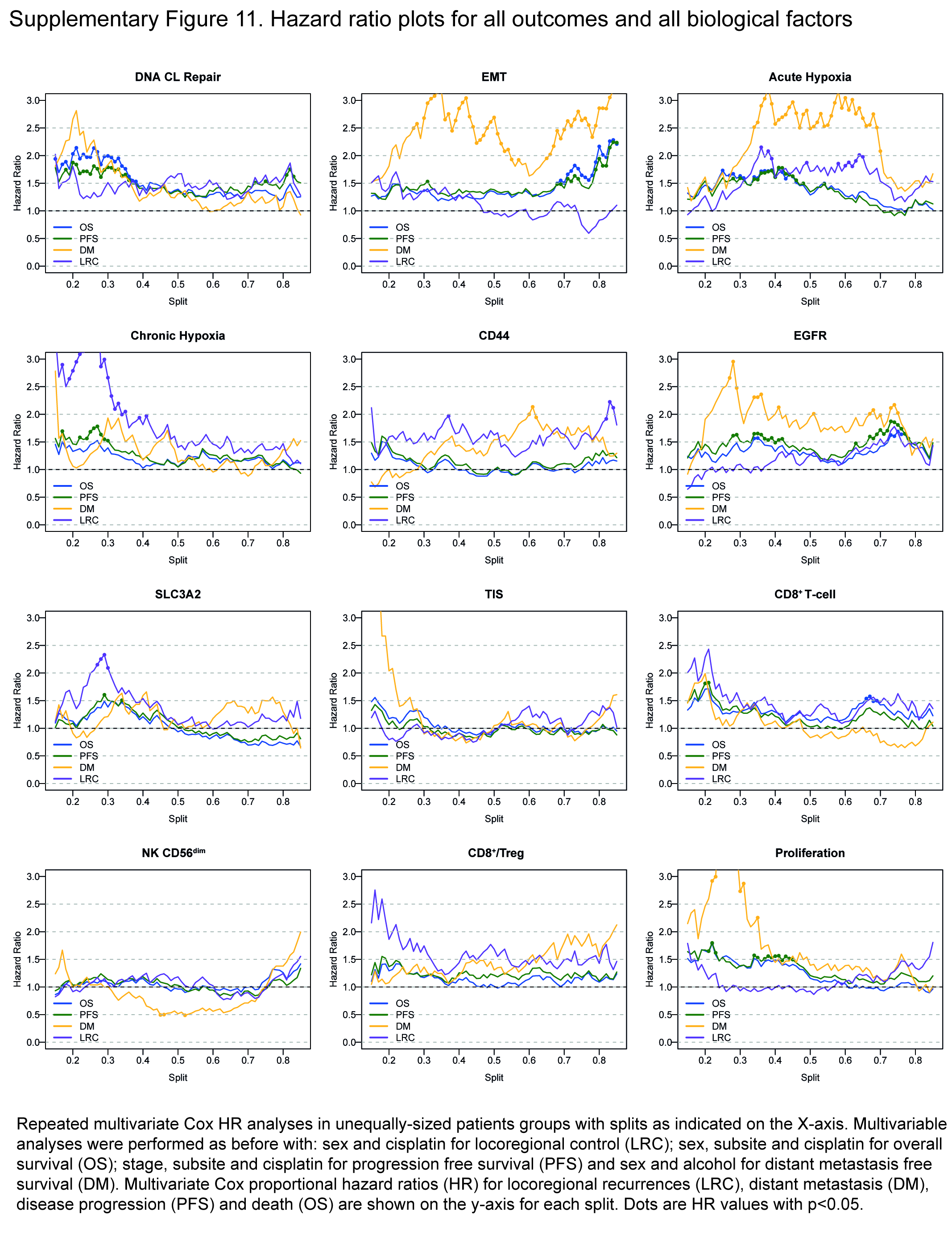

Supplement: Supplementary file 11 [file Image_11.tif]
