# Supplementary material for: Biological Determinants of Chemo-Radiotherapy Response in HPV-Negative Head and Neck Cancer: A Multicentric External Validation
Source: Front Oncol. 2020 Jan 10;9:1470. doi: 10.3389/fonc.2019.01470 (PMC6966332; doi:10.3389/fonc.2019.01470)
Supplement: Supplementary file 12 [file Table_1.PDF]

Supplementary Table 1. Univariate Cox proportional hazard analyses for all clinical variables considered in this study.

REF: reference group

**Variable**			**Locoregional Regression**		**Mortality**		**Progression**		**Distant Metastasis**	
		**N**	HR (95% CI)	*p* -value	HR (95% CI)	*p* -value	HR (95% CI)	*p* -value	HR (95% CI)	*p* -value
Age at diagnosis	≥65	55	0.749 (0.38-1.47)	0,4	1.176 (0.77-1.8)	0,45	0.984 (0.65-1.49)	0,94	0.844 (0.4-1.78)	0,66
	<65	142	REF		REF		REF		REF	
Sex	female	55	0.515 (0.25-1.06)	0,072	0.65 (0.42-1.01)	0,055	0.729 (0.48-1.1)	0,13	**0.334 (0.13-0.85)**	**0,022**
	male	142	REF		REF		REF		REF	
Alcohol	no	22	1.235 (0.52-2.95)	0,64	0.769 (0.4-1.49)	0,44	0.813 (0.43-1.52)	0,52	0.243 (0.03-1.79)	0,17
	former alcoholic	22	**2.158 (1.03-4.52)**	**0,041**	**1.76 (1.05-2.95)**	**0,032**	1.59 (0.95-2.66)	0,076	**2.68 (1.25-5.73)**	**0,011**
	yes	146	REF		REF		REF		REF	
	missing	7								
Tobacco	never	5	0 (0-Inf)	1	0.724 (0.18-2.94)	0,65	0.575 (0.14-2.33)	0,44	0 (0-Inf)	1
	former smoker	30	0.463 (0.17-1.29)	0,14	0.657 (0.37-1.16)	0,15	0.594 (0.34-1.05)	0,071	0.65 (0.23-1.83)	0,42
	yes	156	REF		REF		REF		REF	
	missing	6								
Tumour subsite	Larynx	34	1.47 (0.73-2.96)	0,28	0.615 (0.35-1.09)	0,094	0.822 (0.49-1.38)	0,46	1.026 (0.47-2.25)	0,95
	Hypopharynx	78	0.708 (0.37-1.37)	0,3	**0.517 (0.34-0.78)**	**0,0017**	**0.555 (0.37-0.83)**	**0,0039**	**0.436 (0.2-0.93)**	**0,032**
	Oropharynx	85								
Tumour volume	≥25.4 cc	70	1.754 (0.96-3.19)	0,066	**2.147 (1.42-3.24)**	**0,00027**	**1.832 (1.23-2.72)**	**0,0027**	1.513 (0.76-3)	0,23
	<25.4 cc	96	REF		REF		REF		REF	
	missing	31								
Stage	IVB	20	0.896 (0.35-2.28)	0,82	**1.803 (1.06-3.06)**	**0,029**	1.438 (0.85-2.42)	0,17	2.093 (0.95-4.6)	0,066
	III	40	0.443 (0.19-1.05)	0,064	0.607 (0.36-1.03)	0,063	**0.521 (0.31-0.88)**	**0,014**	0.306 (0.09-1.01)	0,051
	II	2	0 (0-Inf)	1	1.133 (0.16-8.17)	0,9	0.819 (0.11-5.9)	0,84	0 (0-Inf)	1
	IVA	135	REF		REF		REF		REF	
Cumulative cisplatin dose	<200mg/cm2	67	**2.569 (1.45-4.56)**	**0,0012**	**1.737 (1.18-2.55)**	**0,0049**	**1.704 (1.18-2.47)**	**0,0048**	1.175 (0.6-2.29)	0,64
	≥200mg/cm2	126	REF		REF		REF		REF	
	missing	4								
