# Supplementary material for: Biological Determinants of Chemo-Radiotherapy Response in HPV-Negative Head and Neck Cancer: A Multicentric External Validation
Source: Front Oncol. 2020 Jan 10;9:1470. doi: 10.3389/fonc.2019.01470 (PMC6966332; doi:10.3389/fonc.2019.01470)
Supplement: Supplementary file 13 [file Table_2.PDF]

Supplementary Table S2. Cut-offs as determined by the bootstrap method.

Marker	Outcome	Cutoff	fraction(p<0.05)
DNA CL repair	Overall Survival	0,298	0,796
	Progression Free Survival	0,299	0,703
	Locoregional Control	0,348	0,368
	Distant Metastasis	0,276	0,341
EMT	Overall Survival	0,741	0,901
	Progression Free Survival	0,741	0,916
	Locoregional Control	0,015	0,177
	Distant Metastasis	0,053	0,849
Acute Hypoxia	Overall Survival	-0,189	0,707
	Progression Free Survival	-0,186	0,745
	Locoregional Control	-0,018	0,593
	Distant Metastasis	-0,046	0,705
Chronic Hypoxia	Overall Survival	-0,545	0,355
	Progression Free Survival	-0,524	0,655
	Locoregional Control	-0,545	0,870
	Distant Metastasis	0,516	0,205
CD44	Overall Survival	150,247	0,228
	Progression Free Survival	150,247	0,284
	Locoregional Control	424,349	0,548
	Distant Metastasis	278,756	0,489
EGFR	Overall Survival	154,421	0,620
	Progression Free Survival	148,863	0,780
	Locoregional Control	36,609	0,469
	Distant Metastasis	148,863	0,506
SLC3A2	Overall Survival	112,566	0,411
	Progression Free Survival	109,439	0,482
	Locoregional Control	108,867	0,568
	Distant Metastasis	172,793	0,255
TIS	Overall Survival	-0,358	0,240
	Progression Free Survival	-0,358	0,179
	Locoregional Control	-0,035	0,155
	Distant Metastasis	0,186	0,265
$CD8^+$ T cells	Overall Survival	0,002	0,556
	Progression Free Survival	-0,334	0,509
	Locoregional Control	-0,334	0,334
	Distant Metastasis	-0,342	0,125
NK $CD56^{dim}$	Overall Survival	0,341	0,286
	Progression Free Survival	0,358	0,224
	Locoregional Control	-0,501	0,409
	Distant Metastasis	0,308	0,579
$CD8^+/T_{reg}$	Overall Survival	0,580	0,226
	Progression Free Survival	1,649	0,334
	Locoregional Control	1,048	0,403
	Distant Metastasis	4,869	0,387
Proliferation	Overall Survival	-0,495	0,468
	Progression Free Survival	-0,305	0,563
	Locoregional Control	0,476	0,492
	Distant Metastasis	-0,423	0,674
Tumor Volume	Overall Survival	27,500	0,923
	Progression Free Survival	70,700	0,627
	Locoregional Control	26,096	0,284
	Distant Metastasis	70,700	0,324
