# Supplementary material for: Biological Determinants of Chemo-Radiotherapy Response in HPV-Negative Head and Neck Cancer: A Multicentric External Validation
Source: Front Oncol. 2020 Jan 10;9:1470. doi: 10.3389/fonc.2019.01470 (PMC6966332; doi:10.3389/fonc.2019.01470)
Supplement: Supplementary file 14 [file Table_3.PDF]

Supplementary Table S3. Multivariate Cox proportional hazard analysis results of individual markers.

Multivariable analyses used cutoffs as listed in Supplementary Table S2 for each marker and incorporated all clinical variables that resulted to be significantly associated with the respective outcome endpoint.

Corresponding Kaplan Meier Curves are shown in Figure 2 and 3 and Supplementary Figure S5-S7

REF: reference group

**Variable**		**Locoregiol Control**			**Overall Survival**			**Progression**			**DistantMetastasis**		
	Value	N	HR (95% CI)	*p* -value	N	HR (95% CI)	*p* -value	N	HR (95% CI)	*p* -value	N	HR (95% CI)	*p* -value
DNA CL Repair	High	101	1.67 (0.93-3.01)	0,087	136	**1.97 (1.24-3.13)**	**0,00426**	135	**1.8 (1.16-2.79)**	**0,00847**	154	2.69 (0.95-7.61)	0,0628
	Low	96	REF		61	REF		62	REF		43	REF	
EMT	High	154	1.65 (0.77-3.55)	0,196	26	**2.15 (1.32-3.48)**	**0,00202**	26	**2.19 (1.35-3.53)**	**0,00138**	131	**3.14 (1.34-7.39)**	**0,00861**
	Low	43	REF		171	REF		171	REF		66	REF	
Acute Hypoxia	High	73	**1.9 (1.07-3.4)**	**0,0296**	118	**1.62 (1.07-2.47)**	**0,0231**	117	**1.64 (1.09-2.46)**	**0,017**	79	**2.44 (1.25-4.74)**	**0,00857**
	Low	124	REF		79	REF		80	REF		118	REF	
Chronic Hypoxia	High	146	**3.95 (1.56-10.01)**	**0,00381**	146	1.48 (0.94-2.33)	0,0913	143	**1.7 (1.11-2.62)**	**0,0152**	33	1.57 (0.68-3.64)	0,29
	Low	51	REF		51	REF		54	REF		164	REF	
CD44	High	31	**2.03 (1.02-4.04)**	**0,0426**	158	1.46 (0.88-2.41)	0,143	158	1.52 (0.92-2.51)	0,0999	79	**1.97 (1.02-3.8)**	**0,0447**
	Low	166	REF		39	REF		39	REF		118	REF	
EGFR	High	174	0.48 (0.22-1.03)	0,0585	50	**1.68 (1.11-2.53)**	**0,014**	52	**1.82 (1.21-2.72)**	**0,00378**	52	**2.07 (1.07-4.01)**	**0,0317**
	Low	23	REF		147	REF		145	REF		145	REF	
SLC3A2	High	141	**2.31 (1.11-4.82)**	**0,0257**	133	**1.46 (0.96-2.22)**	**0,0791**	137	**1.53 (1.01-2.34)**	**0,0468**	68	1.62 (0.83-3.15)	0,157
	Low	56	REF		64	REF		60	REF		129	REF	
TIS	High	79	1.4 (0.79-2.49)	0,248	167	1.48 (0.82-2.66)	0,188	167	1.32 (0.76-2.3)	0,322	28	1.82 (0.83-4)	0,136
	Low	118	REF		30	REF		30	REF		169	REF	
$CD8^+$ T-cells	High	156	2.35 (0.93-5.97)	0,0715	65	1.58 (1.07-2.32)	0,0208	156	**1.71 (1.01-2.91)**	**0,0473**	158	1.99 (0.7-5.63)	0,196
	Low	41	REF		132	REF		41	REF		39	REF	
NK $CD56^{dim}$	High	174	0.5 (0.22-1.13)	0,0967	27	1.45 (0.88-2.38)	0,142	26	1.31 (0.79-2.17)	0,302	30	**2.47 (1.16-5.24)**	**0,0188**
	Low	23	REF		170	REF		171	REF		167	REF	
$CD8^+/T_{reg}$	High	97	1.55 (0.86-2.79)	0,141	152	1.37 (0.84-2.22)	0,21	75	1.34 (0.92-1.94)	0,123	30	2 (0.94-4.25)	0,0732
	Low	100	REF		45	REF		122	REF		167	REF	
Proliferation	High	26	1.89 (0.96-3.75)	0,0668	155	1.62 (0.96-2.74)	0,07	119	**1.5 (1.02-2.21)**	**0,0418**	140	**3.61 (1.27-10.31)**	**0,0162**
	Low	171	REF		42	REF		78	REF		57	REF	
