# Supplementary material for: Biological Determinants of Chemo-Radiotherapy Response in HPV-Negative Head and Neck Cancer: A Multicentric External Validation
Source: Front Oncol. 2020 Jan 10;9:1470. doi: 10.3389/fonc.2019.01470 (PMC6966332; doi:10.3389/fonc.2019.01470)
Supplement: Supplementary file 15 [file Table_4.PDF]

Supplementary Table S4. Multivariate Cox proportional hazard analysis results.

Multivariable analyses incorporated all clinical and biological variables remaining after the backward selection procedure.

Outcome	variable	HazardRatio	*p* -value
Mortality	Sex: female	0.53 (0.3-0.94)	**0.03**
	Tobacco: former-smoker	0.61 (0.31-1.21)	0.16
	Tobacco: never	0 (0-Inf)	1
	Stage: III	0.64 (0.33-1.22)	0.17
	Stage: IVB	2.47 (1.2-5.05)	**0.014**
	Cisplatin: Low	2.57 (1.6-4.13)	**1,00E-04**
	DNA CL Repair: Low	0.47 (0.28-0.81)	**0.0058**
	EMT: High	2.42 (1.35-4.34)	**0.0029**
	EGFR: High	1.7 (1.06-2.72)	**0.028**
	SLC3A2: Low	0.57 (0.35-0.93)	**0.025**
	NK $CD56^{dim}$: High	1.86 (1.08-3.2)	**0.024**
Progression	Sex: female	0.6 (0.34-1.05)	0.071
	Tobacco: former-smoker	0.53 (0.27-1.05)	0.067
	Tobacco: never	0 (0-Inf)	1
	Stage: III	0.77 (0.4-1.49)	0.44
	Stage: IVB	1.98 (0.97-4.07)	0.062
	Cisplatin: Low	2.79 (1.73-4.51)	**2.8e-05**
	DNA CL Repair: Low	0.52 (0.31-0.88)	**0.015**
	EMT: High	2.4 (1.31-4.38)	**0.0044**
	Acute Hypoxia: Low	0.62 (0.36-1.08)	0.092
	Chronic Hypoxia: Low	0.57 (0.33-0.98)	**0.041**
	EGFR: High	1.55 (0.97-2.48)	0.069
	SLC3A2: Low	0.65 (0.38-1.11)	0.11
	$CD8^+$ T-cells: Low	0.61 (0.31-1.2)	0.15
	NK $CD56^{dim}$: High	1.68 (0.95-2.97)	0.074
	Proliferation: Low	1.71 (0.96-3.04)	0.068
	Tumor Volume: High	1.78 (0.99-3.2)	0.055
Locoregional Recurrences	Age Over 65	0.34 (0.15-0.74)	**0.007**
	Sex: female	0.24 (0.1-0.59)	**0.002**
	Cisplatin: Low	3.07 (1.59-5.93)	**0.00084**
	Chronic Hypoxia: Low	0.23 (0.08-0.68)	**0.0073**
	CD44: High	1.92 (0.89-4.13)	0.097
	EGFR: Low	2.89 (1.21-6.89)	**0.017**
	TIS: High	2.21 (1.05-4.65)	**0.036**
	$CD8^+/T_{reg}$: High	2.62 (1.26-5.45)	**0.01**
Distant Metastasis	Sex: female	0.27 (0.07-1)	0.05
	Alcohol: former-Alcohol: ic	2.61 (1.04-6.56)	**0.041**
	Alcohol: no	0.31 (0.04-2.48)	0.27
	Stage: III	0.31 (0.07-1.41)	0.13
	Stage: IVB	5.22 (1.74-15.69)	**0.0032**
	Subsite: Hypopharynx	0.37 (0.14-0.97)	**0.043**
	Subsite: Larynx	1.2 (0.47-3.07)	0.7
	EMT: Low	0.23 (0.08-0.6)	**0.0028**
	EGFR: High	3.19 (1.4-7.23)	**0.0056**
	TIS: High	2.37 (0.94-5.97)	0.066
	$CD8^+/T_{reg}$: High	2.94 (1.14-7.53)	**0.025**
	Proliferation: Low	0.29 (0.07-1.25)	0.097
